# Effects and Safety of Non-Pharmacological Therapies of Traditional Chinese Medicine for Coronary Heart Disease: An Overview of Systematic Reviews

**DOI:** 10.1155/2022/8465269

**Published:** 2022-03-19

**Authors:** Weiqiang Ji, Lan Wu, Guangming Pan, Xu Zou

**Affiliations:** ^1^The Second School of Clinical Medicine, Guangzhou University of Chinese Medicine, Guangzhou, Guangdong, China; ^2^Nanfang Hospital, Southern Medical University, Guangzhou, Guangdong, China; ^3^Guangdong Provincial Hospital of Chinese Medicine, The Second Affiliated Hospital of Guangzhou University of Chinese Medicine, Guangzhou, Guangdong, China

## Abstract

**Objectives:**

Coronary heart disease (CHD) is currently the leading cause of human death. Non-pharmacological therapy of traditional Chinese medicine (NPTCM) is an important characteristic therapy of traditional Chinese medicine (TCM). Questions concerning the efficacy and safety of NPTCM-related interventions in patients with CHD led us to conduct this overview of systematic reviews (SRs).

**Methods:**

The Cochrane Library, PubMed, Embase, EBSCO, Epistemonikos, PROSPERO, CNKI, Wanfang Database, and SinoMed were searched without language and publication status restriction from their inception to May 2021, along with grey literature sites and reference lists of included reviews. Systematic reviews comparing NPTCM/a combination of NPTCM and non-TCM interventions with non-TCM interventions/inactive controls for CHD were examined. Two reviewers independently screened titles, abstracts, and full-text articles, and completed data extraction and quality appraisal according to the predefined standards.

**Results:**

In total, 1494 titles and abstracts and 66 full-text articles were screened, and a total of 12 SRs (11 with meta-analysis) were finally included. According to PRISMA 2020 checklist, more than 50% of reviews conformed to 80% of 54 items. Consistent evidence of effectiveness or harms across multiple outcomes based on more than one moderate quality review with meta-analysis was found for acupuncture and usual care plus acupuncture/Baduanjin/TCM exercise therapies/TCM emotional therapy. These interventions were mostly evaluated less than 6 months.

**Conclusion:**

Acupuncture or acupuncture plus usual care could improve angina symptoms and ECG, and usual care plus Baduanjin/TCM exercise therapies could ameliorate health-related quality of life. Additionally, Baduanjin plus usual care could also improve psychological condition, and it as well as acupuncture could be safe due to no reports on adverse events related to these interventions. TCM emotional therapy plus usual care could benefit patients with CHD and depression.

## 1. Introduction

Coronary heart disease (CHD) is one of the most common cardiovascular diseases and the main cause of death in humans [1]. The mortality rate of CHD in Chinese urban residents was 120.18/100,000 in 2018 and the number of patients with this disease is now more than 11 million. With the development of global economy and society and the increase of the elderly population, the risk factors of cardiovascular disease also increase. Although medical therapies for CHD have made great improvement in recent years, the prevalence and mortality of this disease are still on the upward trend in China [2].

Non-pharmacological therapies of traditional Chinese medicine (NPTCM), including acupuncture therapy, Tai Chi, qigong, Baduanjin, and psychological intervention and music therapy, is an important characteristic therapy of traditional Chinese medicine (TCM) and has been implemented to treat CHD for about 2,000 years because of its simpleness, convenience, cheapness, and effectiveness. According to the Huangdi Neijing, written more than 2,000 years ago, acupuncture therapy could be used for CHD [3]. NPTCM may play an important role in relieving angina symptoms and improving myocardial ischemia, especially in the rehabilitation of patients with CHD [4, 5]. In recent years, although there has been an increasing number of systematic reviews (SRs) about NPTCM for CHD, the effectiveness and safety of NPTCM may still need to be evaluated due to their various types and roles. In addition, there is still a lack of evaluation of SRs about NPTCM for CHD.

Therefore, the aim of this study is to conduct an overview of SRs to evaluate the safety and effectiveness of NPTCM/a combination of NPTCM and non-TCM interventions versus non-TCM interventions/inactive controls (e.g., sham acupuncture, wait-list control, etc.) for CHD, along with the methodological and report quality of included reviews.

## 2. Methods

### 2.1. Protocol Registration

The protocol of this overview was registered with the International Prospective Register of Systematic Reviews database (registration number: CRD42021269760; https://www.crd.york.ac.uk/prospero/). This overview was performed following the Preferred Reporting Items for OoSRs (PRIO-harms) checklist (Supplementary Material 1) [6].

### 2.2. Eligibility Criteria

The inclusion criteria for the overview were established to include the following:

- Study design: systematic knowledge syntheses including primary studies of randomized controlled trials (RCTs) with or without a meta-analysis.
- Patients: without limitation of race, age, sex, and disease course; patients were diagnosed with CHD (acute or previous myocardial infarction, stable or unstable angina) according to the definitions used in individual studies.
- Interventions: NPTCM interventions included acupuncture therapy, Tai Chi, qigong, Baduanjin, TCM psychological intervention, and TCM music therapy, etc.; or a combination of NPTCM and non-TCM interventions.
- Comparator: inactive controls (e.g., sham acupuncture, wait-list control, etc.) or active controls (non-TCM interventions).
- The outcomes are as follows:
- *Effectiveness*. All-cause mortality; cardiovascular mortality; composite cardiovascular events (CCEs), including cardiovascular death, non-fatal myocardial infarction, unstable angina pectoris, resuscitated cardiac arrest, stroke, and cardiac revascularization procedures; cardiovascular-related hospital admissions; clinical effects including total clinical efficiency, time to onset of angina relief in response to treatment and improvements in angina symptoms or electrocardiograph (ECG); health-related quality of life including Seattle Angina Questionnaire (SAQ), 36-Item Short Form Health Survey (SF-36), and Minnesota Living With Heart Failure Questionnaire (MLHFQ); psychological assessment including Self-Rating Depression Scale (SDS), Self-Rating Anxiety Scale (SAS), and Hamilton Depression Scale (HAMD); exercise tolerance assessment including 6-minute walking test (6MWT), metabolic equivalents (METs), peak oxygen consumption (VO_2_peak), and oxygen consumption/heart rate (VO_2_/HR).
- *Harms*. Adverse events related to NPTCM interventions.

The exclusion criteria for the overview were established to include the following: repeated published literature or literature that cannot be obtained full text; network meta-analysis (NMA); protocol of SRs; ongoing SRs; interventions included TCM, Chinese patent medicine, and the comparators were other NPTCM; articles identified as rapid reviews, literature reviews, and narrative reviews; interventions were NPTCM along with non-TCM treatment, but controls did not give the corresponding non-TCM treatment; SRs included non-RCT studies.

### 2.3. Literature Search

The Cochrane Database of Systematic Reviews (https://www.cochranelibrary.com), PubMed (https://pubmed.ncbi.nlm.nih.gov), Embase (https://www.embase.com), EBSCO (AMED, CINAHL, PsycINFO; https://www.ebsco.com), Epistemonikos (https://www.epistemonikos.org), the PROSPERO register (https://www.crd.york.ac.uk/PROSPERO), Chinese National Knowledge Infrastructure (CNKI; https://www.cnki.net), Wanfang Database (https://www.wanfangdata.com.cn), and SinoMed (https://www.sinomed.ac.cn) were searched without language and publication status restriction from their inception to May 2021. The search strategy contained both MeSH terms and relevant keywords, and a methodological filter was used to limit the search to SRs and meta-analyses. The search terms including the following: coronary disease, coronary heart disease, angina pectoris, myocardial infarction, acupuncture therapy, ear acupuncture, electroacupuncture, moxibustion, massage, cupping therapy, Taiji, qi gong, Baduanjin, psychotherapy, music therapy, SR, meta-analysis, etc. We also searched grey literature sites including Google Scholar (https://scholar.google.com), GreyNet International (https://www.greynet.org), and Grey Literature Report (https://www.greylit.org). In addition, we reviewed reference lists of included reviews and tried to contact the authors of relevant review protocols for their manuscripts or unpublished data. The full search strategy is available in Supplementary Materials 2: Appendix A.

### 2.4. Study Selection and Data Extraction

Two reviewers (WJ and LW) independently screened literature and extracted data, and any discrepancies were resolved by a third reviewer (GP). The missing data in related reviews were available via contacting with the author as far as possible. Titles and abstracts were screened prior to full-text screening, and then eligible reviews were included. The data item extracted from reviews included the first author's name, the year of publication, the author's country, patient characteristics (e.g., type, age, and number of patients), the number of included studies, funding, type and course of intervention, evaluation tools for quality of studies, outcome extracted (e.g., name, definition, and results of statistical analysis of outcome), and methodological and report quality of SRs.

### 2.5. Quality Appraisal and Assessment of Evidence

The quality appraisal of methodology and report along with assessment of evidence quality were conducted concurrently with data extraction respectively using the Assessing the Methodological Quality of Systematic Reviews tool version 2 (AMSTAR 2) [7], the Preferred Reporting Items for Systematic reviews and Meta-Analyses (PRISMA) 2020 checklist [8], and the Grading of Recommendations Assessment, Development, and Evaluation (GRADE) [9]. The assessments were completed independently by two reviewers and any discrepancies were resolved by a third reviewer.

The PRISMA 2020 checklist including 7 sections with 27 items replaces the 2009 checklist and the structure along with presentation of its items have been modified to facilitate implementation. The PRISMA 2020 for Abstracts checklist, which consists of 12 items and is an update of the 2013 PRISMA for Abstracts checklist, was implemented to assess abstracts of included reviews.

In the GRADE algorithm, each review begins with a ranking of high certainty and is downgraded 1 level for serious methodological limitations (number of participants within pooled analysis between 100 and 199; low risk of bias in randomization and blinding for <75% included studies; high heterogeneity (*I*^2^ > 75%); and “No” on one of these AMSTAR 2 items: a priori research design, comprehensive literature search, duplicate study selection, or duplicate data extraction) or 2 levels for very serious limitations (number of participants within pooled analysis <100 and “No” on two or more of these AMSTAR 2 items: a priori research design, comprehensive literature search, duplicate study selection, or duplicate data extraction) [9].

### 2.6. Data Synthesis

Since significant heterogeneity was expected in clinical characteristics and methodological quality across the included reviews, performing formal statistical analysis (e.g., pooling data or conducting an indirect comparison) would not be appropriate under this condition. We generated citation matrices that cross-linked individual reviews with all of included primary studies. In addition, the “corrected covered area” (CCA) was calculated to quantify the degree of overlap across all reviews included in this overview [10].

## 3. Results

### 3.1. Literature Search

**Figure 1:**
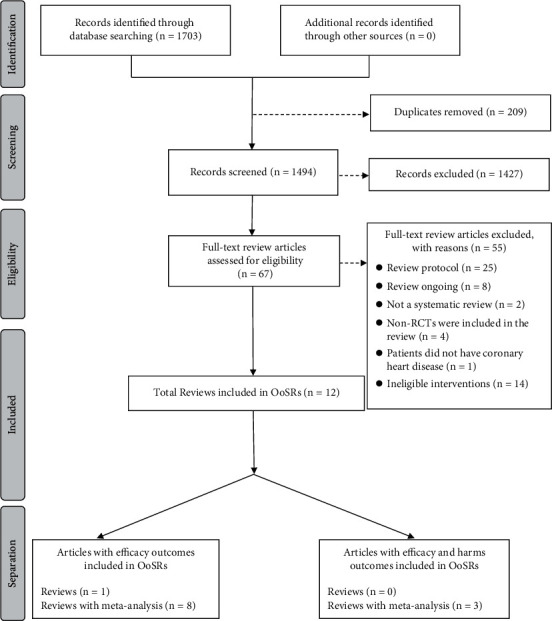
Flow chart for overview of systematic reviews (OoSRs).

A total of 1703 articles were initially obtained according to our predefined search strategy. Sixty-seven full-text articles were retrieved after de-duplication and titles/abstracts screening, 55 of which were excluded for not meeting eligibility criteria and 12 of which were included in this overview and eligible for data extraction (Figure 1). A total of 71 primary studies were cited 99 times across the 11 SR + MAs [11–21] and 1 SR [22] included in this overview, resulting in a CCA of 0.036 indicating little overlap across included reviews.

### 3.2. Review and Study Characteristics

The included reviews were performed between 2012 and 2021 with the majority (92%) published after 2014. A total of 11 published reviews and one unpublished review were contained in the reviews included, 10 of which in Chinese and 2 in English. As to the evaluation of methodological quality of included RCTs, 9 reviews implemented the evaluation tool with regarding to risk of bias recommended by the Cochrane Library while 3 reviews used Jadad scale or modified Jadad scale.

The overall sample size of included reviews averaged 690 patients (range 20–1267). Other participant characteristics such as mean age and the percentage of female patients were reported in only 3 SR + MAs and 1 SR. The included reviews examined a total of 11 distinct treatment comparisons across 5 different types of NPTCM interventions. The number of included reviews reporting duration of each treatment, frequency of the intervention, total duration of treatment, and length of follow-up were 4, 5, 10, and 2, respectively. Relevant 12 reviews that examined at least one eligible intervention could be identified for the effectiveness outcomes including CCEs, clinical effects, health-related quality of life, psychological assessment, exercise tolerance assessment. A total of 3 relevant reviews reported the harms such as adverse events related to NPTCM interventions. Basic characteristics of included reviews and studies are presented in Table 1.

### 3.3. Quality Appraisal and Assessment of Evidence

**Figure 2:**
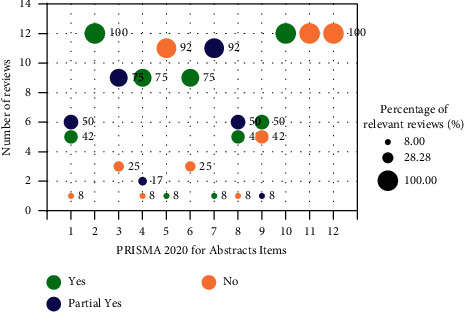
PRISMA 2020 for Abstracts results.

**Figure 3:**
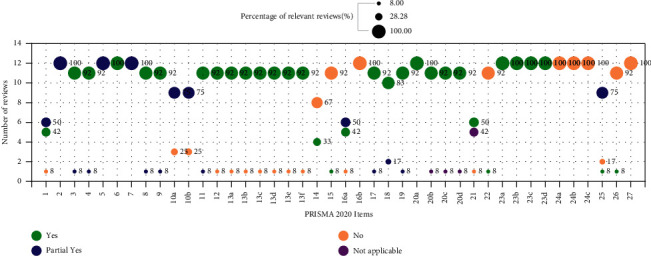
PRISMA 2020 results.

According to PRISMA 2020 checklist, the majority of included reviews (≥75%) reported the items of objectives, information sources, synthesis of results, and interpretation in their abstracts, but none of relevant reviews reported the funding and registration information in their abstracts and full texts. In addition, there were over 92% of included reviews reporting the items of rationale and objectives in introduction, information sources, selection process, data collection process, study risk of bias assessment, effect measures and synthesis methods in methods, study characteristics, results of individual studies and results of syntheses in results, and discussion confirming to the PRISMA 2020. Additionally, few of included reviews (less than 8%) reported items of certainty assessment in methods, certainty of evidence in results, and competing interests in other information, and none of relevant reviews reported the items of study selection (16b) in results, registration and protocol, and availability of data, code, and other materials in other information. The full PRISMA results are available in Figures 2 and 3 and Supplementary Materials 2: Appendix B.

**Figure 4:**
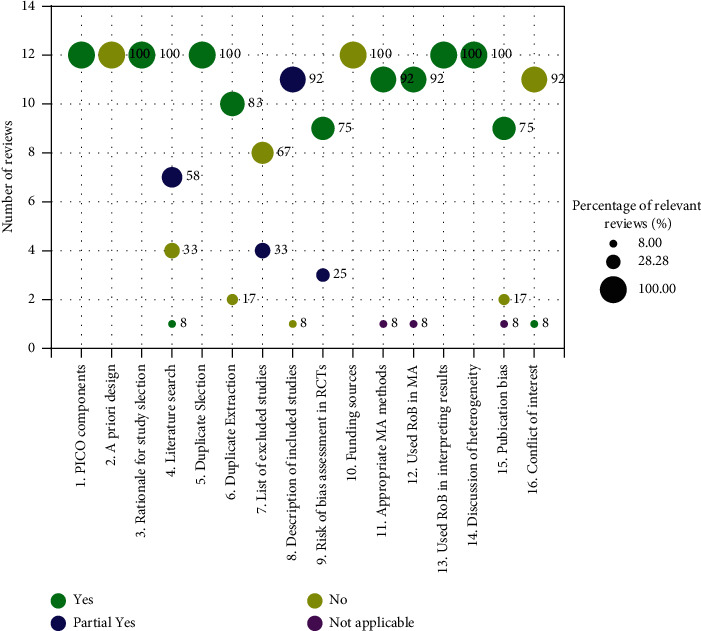
AMSTAR 2 results.

The included reviews were rated as low quality (2 SR + MAs, 17%; 1 SRs, 8%), or critically low quality (9 SR + MAs, 75%; Figure 4). Specifically, out of the 16 individual items, 5 was fully reported (PICO components, rationale for study selection, duplicate selection, used RoB in interpreting results and discussion of heterogeneity). However, none of the included reviews explicitly stated that the following two items: a priori design and funding sources. The full AMSTAR 2 results are available in Supplementary Materials 2: Appendix C.

Out of the 9 types of interventions included in this overview, only 5 comparisons (acupuncture, acupuncture plus usual care, TCM exercise therapies plus usual care, Baduanjin plus usual care, and TCM emotional therapy plus usual care (all compared usual care)) included reviews rated with a moderate strength of evidence based on GRADE, and 4 comparisons (Taiji plus usual care; Tai Chi Chuan compared to physical activity counseling; Taiji plus jogging compared to jogging; and Baduanjin compared to usual medical exercise) included reviews rated with a low or very low strength of evidence (Table 2). None of the comparisons included in this overview included reviews rated as having a high strength of evidence based on GRADE (Table 2).

### 3.4. Outcome Results

#### 3.4.1. Acupuncture

One low-quality [19] and two critically low-quality [18, 20] SR + MA compared acupuncture plus usual care to usual care and found statistically significant reduction in CCEs (3 RCTs and 207 patients) and greater improvements in clinical effects (improvement in angina symptoms; 22 RCTs and 1644 patients), clinical effects (improvement in ECG; 19 RCTs and 1401 patients), and clinical effects (time to onset of angina relief in response to treatment; 2 RCTs and 162 patients). One low-quality [19] and one critically low-quality [20] SR + MA compared acupuncture plus usual care to usual care and found none of the studies reported any adverse effects associated with acupuncture therapy (19 RCTs and 1543 patients). One critically low-quality [20] SR + MA compared acupuncture to usual care and found statistically significant improvements in clinical effects (improvement in angina symptoms; 2 RCTs and 206 patients) and clinical effects (improvement in ECG; 2 RCTs and 206 patients) but found no statistically significant difference in the improvement in clinical effects (time to onset of angina relief in response to treatment; 1 RCT and 128 patients), and they also found none of the studies reported any adverse effects associated with acupuncture therapy (2 RCTs and 206 patients). One critically low-quality [16] SR + MA compared acupuncture plus usual care to usual care and found statistically significant improvements in clinical effects (total clinical efficiency; 2 RCTs and 152 patients). One critically low-quality [16] SR compared acupuncture plus usual care to usual care and found statistically significant improvements in clinical effects (improvement in angina symptoms; 1 RCT and 30 patients) and SAQ (total score; 1 RCT and 30 patients) but found no significant improvements in clinical effects (improvement in ECG; 1 RCT and 63 patients), SAS (1 RCT and 30 patients) and SDS (1 RCT and 30 patients).

#### 3.4.2. Baduanjin

Two critically low-quality [11, 15] SR + MA compared Baduanjin plus usual care to usual care and found statistically significant improvements in 6MWT (5 RCTs and 474 patients). One critically low-quality [15] SR + MA compared Baduanjin plus usual care to usual care and found statistically significant improvements in METs (3 RCTs and 232 patients). One low-quality [12] and two critically low-quality [11, 14] SR + MA compared Baduanjin plus usual care to usual care and found statistically significant improvements in SAS (8 RCTs and 870 patients) and SDS (6 RCTs, 650 patients). One low-quality [12] and one critically low-quality [14] SR + MA compared Baduanjin plus usual care to usual care and found statistically significant improvements in SAQ (physical limitation; 8 RCTs and 517 patients), SAQ (angina frequency; 9 RCTs and 817 patients), SAQ (angina stability; 9 RCTs and 817 patients), and SAQ (disease perception; 9 RCTs and 817 patients) and no significant difference (4 RCTs and 310 patients) or greater improvement (9 RCTs and 817 patients) in SAQ (treatment satisfaction). One critically low-quality [14] SR + MA compared Baduanjin plus usual care to usual care and found no adverse events related to Baduanjin in the intervention group across included studies (13 RCTs and 1206 patients). One RCT (60 patients) [23] included in one low-quality [12] and one critically low-quality [14] SR + MA compared Baduanjin to usual medical exercise and found statistically significant improvements in SAQ (physical limitation) and SAQ (treatment satisfaction) and no significant difference in SAQ (angina frequency), SAQ (angina stability), SAQ (disease perception), and adverse events related to the intervention.

#### 3.4.3. Taiji or Tai Chi Chuan

One critically low-quality [17] SR + MA compared Taiji plus usual care to usual care and found statistically significant improvements in SF-36 (1 RCT and 60 patients) and MLHFQ (1 RCT and 132 patients). One critically low-quality [17] SR + MA compared Taiji plus jogging to jogging and found statistically significant improvement in SF-36 (1 RCT and 60 patients). One critically low-quality [22] SR compared Tai Chi Chuan to physical activity counseling and found no statistically significant difference in improvement in VO_2_peak (1 RCT, 20 patients).

#### 3.4.4. TCM Exercise Therapies

One critically low-quality [13] SR compared TCM exercise therapies plus usual care to usual care and found more si*gnificant reduction in the incidence of CCEs (1 RCT and 1*00 patients). One critically low-quality [13] SR + MA compared TCM exercise therapies plus usual care to usual care and found statistically significant improvements in clinical effects (improvement in angina symptoms; 2 RCTs and 282 patients), VO_2_/HR (2 RCTs and 186 patients), SAS (3 RCTs and 150 patients), SDS (3 RCTs and 150 patients), SAQ (physical limitation; 4 RCTs and 248 patients), SAQ (angina frequency; 4 RCTs and 248 patients), SAQ (angina stability; 4 RCTs and 248 patients), and SAQ (treatment satisfaction; 4 RCTs and 248 patients). In addition, one critically low-quality [13] SR + MA compared TCM exercise therapies plus usual care to usual care and found no statistically significant difference in VO_2_peak (2 RCTs and 186 patients) and SAQ (disease perception; 4 RCTs and 248 patients).

#### 3.4.5. TCM Emotional Therapy

One critically low-quality [21] SR + MA compared TCM emotional therapy plus usual care to usual care and found statistically significant improvements in clinical effects (total clinical efficiency; 7 RCTs and 629 patients), HAMD (4 RCTs and 270 patients), SAS (3 RCTs and 186 patients), and SDS (3 RCTs and 186 patients).

The outcome results are available in Supplementary Materials 2: Tables D1–D5Appendix D. Tables with the overlap in the primary studies included in relevant reviews can be found in Tables E1–E5 Supplementary Materials 2: Appendix E and in Supplementary Materials 3.

## 4. Discussion

Coronary heart disease is a pathological process characterized by atherosclerotic plaque accumulation in the epicardial arteries, whether obstructive or non-obstructive [24]. The incidence of CHD in China is gradually increasing, and its mortality rate accounts for the first place in cardiovascular disease [25]. In recent years, especially the acupuncture therapy, Baduanjin, tai ji, and TCM emotional therapy in NPTCM have been implemented gradually in rehabilitation and treatment of CHD. Acupuncture and Taiji have been widely used in the rehabilitation and treatment of chronic diseases in the world. The application of acupuncture therapy through stimulating certain acupoints on the human body is to activate the meridians and collaterals and to regulate the function of the internal organs, qi, and blood so as to prevent and treat diseases. Acupuncture therapy can especially reinforce the action of the sympathetic nervous system and cerebral cortex which control all the tissues and organs of the human body. Taiji, including low- to moderate-intensity traditional aerobic exercises, essentially involves learning a sequence of movements that can vary according to different styles. Individuals practice Taiji primarily to develop mind-body interaction, breathing and movement control, eye-hand coordination, and a peaceful state of mind [22]. Baduanjin, translated as 8 silken movements and characterized by interplay between symmetrical physical postures and movements, mind, and breathing exercise in a harmonious manner, is one of the most common forms of qigong exercise and an alternative to the most common cardiac rehabilitation programmes due to their scarcity and unaffordability in China [26, 27]. Its primary focus is on the release of internal body energy with the intent of producing diverse health benefits [27]. TCM emotional therapy, based on the restraining and engendering of TCM 5-phase theory (5-element theory), may play an important role in the rehabilitation of CHD due to the high incidence of psychological problems in patients with this disease [28]. Meditation is a state of consciousness in which the individual eliminates environmental stimuli from awareness so that the mind can focus on a single thing, producing a state of relaxation and relief from stress. Although meditation is one of the non-pharmacological therapies, it is most often associated with Buddhism and Hinduism [29] and should not be included in NPTCM due to its different basis theory from TCM theory.

Our comprehensive overview of reviews included 12 SRs representing 71 primary studies and found consistent evidence of effectiveness for NPTCM. Usual care plus acupuncture/TCM exercise therapies compared to usual care could result in statistically significant reduction in the incidence of CCEs. As to clinical effects, statistically significant improvement in the total clinical efficiency appeared in usual care plus acupuncture/TCM emotional therapy compared to usual care; TCM exercise therapies plus usual care compared to usual care could significantly improve angina symptoms; both of acupuncture plus usual care and acupuncture compared to usual care could result in statistically significant improvements in angina symptoms and ECG (ST segment ischemia), and acupuncture plus usual care also resulted in statistically significant reduction in the time to onset of angina relief in response to treatment, while acupuncture did not. Therefore, acupuncture may not be advised for the emergency treatment of ischemic disease. In terms of SAQ, both of Baduanjin plus usual care and TCM emotional therapy plus usual care compared to usual care resulted in greater improvements in SAQ (physical limitation, angina frequency, angina stability, and treatment satisfaction), and the former also significantly improve the SAQ (disease perception) while the latter not; when Baduanjin vs usual medical exercise, greater improvements in SAQ (physical limitation and treatment satisfaction) were observed in Baduanjin, while there was no significant difference in the improvements in SAQ (angina frequency, angina stability, and disease perception). Taiji plus usual care could significantly improve the scores of SF-36 and MLHFQ when compared to usual care. When compared to usual care, usual care plus Baduanjin/TCM exercise therapies/TCM emotional therapy could result in statistically significant improvements in both SAS and SDS, while acupuncture plus usual care did not. In addition, TCM emotional therapy plus usual care compared to usual care could also significantly improve HAMD score. As to exercise tolerance assessment, Baduanjin plus usual care compared to usual care could result in statistically significant improvement in METs and 6MWT, while acupuncture plus usual care did not; when compared to usual care, greater improvement in VO_2_/HR was shown in the intervention of TCM exercise therapies plus usual care, but there was no significant difference in improvement in VO_2_peak in this intervention as well as Taiji compared to physical activity counseling. In terms of adverse events related to the intervention, all of Baduanjin, acupuncture, Baduanjin plus usual care, and acupuncture plus usual care compared to usual care could result in no statistically significant difference.

The included reviews conformed to majority of items in both of PRISMA 2020 checklist and PRISMA 2020 for Abstracts checklist, but there are still limitations of these reviews worth noting, particularly few reviews reported items of certainty of evidence and competing interests, and none of relevant reviews reported the items of registration and protocol, and availability of data, code, and other materials in other information. Systematic reviews in this field could be improved by increasing the reports of these aforementioned items. In addition, there are also some limitations of included reviews that should be taken into consideration since that all of these reviews received a low- or critically low-quality score on the AMSTAR 2 tool. Moreover, none of the comparisons included in this overview included reviews rated as having a high strength of evidence based on GRADE. This suggests that current results should be interpreted with caution and that some improvements are needed in the study design and in the methods implemented to synthesize knowledge in this field.

There are also some limitations to the conduct of this overview that may worth noting. The obtained reviews in this overview were mainly published in China, but some unpublished SRs and studies may appear in other regions. Although we had searched published literature and relevant grey literature comprehensively, there is a relevant review that we could not obtain the full text, of which may have some impact on the comprehensiveness of search results.

There are several strengths of this overview that should be taken into consideration, particularly a study protocol to guide the conduct of the overview, as well as the implementation of the PRISMA 2020, AMSTAR 2, and GRADE tools for quality appraisal. The literature search was comprehensive and included both published and unpublished reviews without limitations of publication date and language. Moreover, all the included reviews were closely examined for overlaps in the primary evidence.

## 5. Conclusions

According to the results of this overview, acupuncture or acupuncture plus usual care could improve angina symptoms and ECG (ST segment ischemia), and it could be safe because that none of adverse events related to acupuncture therapy was reported in this field. Baduanjin plus usual care and TCM exercise therapies plus usual care could be used to treat CHD due to their improvements in health-related quality of life on physical limitation, angina stability, and angina stability in SAQ. Additionally, Baduanjin plus usual care could also improve the disease perception in SAQ and psychological condition in terms of SAS and SDS, and it could be safe due to no reports on adverse events related to this intervention in primary studies. TCM emotional therapy plus usual care could benefit patients with CHD and depression because of the improvement in HAMD. However, these aforementioned NPTCM have only been implemented in short-term studies, and there is little evidence for their effectiveness or safety beyond 6 months of treatment. [29]

## Figures and Tables

**Table 1:** Summary of review and participant characteristics.

Author, publication year	Region	Type of review	Literature search dates	Included primary studies (sample size)	Age [mean (SD)]	Sex (female, %)	Intervention measures		Duration of each treatment (range, minutes)	Frequency	Total duration of treatment (range, weeks)	Length of follow-up (range, weeks)	Outcomes	Assessment tool of RoB in included RCTs
							Intervention group	Control group						
Xu, 2021	China	SR + MA	Inception to January, 2020	9 (985)	NR	NR	Baduanjin plus usual care	Usual care	30 to 45	2 to 7 times/week	8 to 24	NR	6MWT, SAS, and SDS	Recommended by Cochrane Library
Xu, 2020	China	SR + MA	Inception to 28 November, 2019	1 (60)	NR	NR	Baduanjin	Usual medical exercise	NR	Two times/day and 5 days/week	12	12	SAQ	Recommended by Cochrane library
				6 (540)	NR	NR	Baduanjin plus usual care	Usual care	20 to 60	3 to 4 times/week, or 1 to 2 times/day and 3 to 5 times/week	1 week to 6 months	1 week to 6 months	SAS, SDS, and SAQ	
Zhang, 2020	China	SR + MA	Inception to June, 2019	8 (649)	55(8.7)–65(2.2)/40–70(NR)	NR	TCM exercise therapies plus usual care	Usual care	NR	NR	4 to 24	NR	VO_2_peak, VO_2_/HR, SAS, SDS, SAQ, clinical effects^a^, CCEs	Recommended by Cochrane Library
Luo, 2020	China	SR + MA	January., 2009 to December, 2019	1 (60)	NR	23	Baduanjin	Usual medical exercise	NR	NR	12	NR	SAQ, adverse events^b^	Jadad scale
				13(1207)	NR	41(NR in one study)	Baduanjin plus usual care	Usual care	NR	NR	1 month to 6 months	NR	SAS, SDS, SAQ, adverse events^b^	
Yang, 2019	China	SR + MA	Inception to October, 2018	5 (457)	55.34(12.32)–70.3(6.4)	32	Baduanjin plus usual care	Usual care	NR	NR	2 months to 6 months	NR	6MWT, METs	Recommended by Cochrane Library
Zhou, 2018	China	SR + MA	Inception to 20 June, 2017	4 (245)	NR	NR	Acupuncture plus usual care	Usual care	NR	NR	2 to 8	NR	6WMT, SAS, SDS, SAQ, clinical effects^c^	Recommended by Cochrane Library
Chen, 2018	China	SR + MA	Inception to 31 December, 2016	1 (60)	NR	NR	Taiji plus jogging	Jogging	60 minutes for Taiji and 30 minutes for jogging	5 times/week for Taiji and 7 times/week for jogging	6 months	NR	SF-36	Recommended by Cochrane Library
				2 (192)	NR	NR	Taiji plus usual care	Usual care	60 or ≥30	5 times/week	6 months or 12 months	NR	MLHFQ, SF-36	
Li, 2017	China	SR + MA	Inception to 1 July, 2016	7 (465)	NR	NR	Acupuncture plus usual care	Usual care	NR	NR	10 days to 15 days	NR	Clinical effects^d^	Jadad scale
Li, 2016	China	SR + MA	Inception to 1 December, 2015	14 (1164)	45(NR)- 85(NR)	NR	TCM emotional therapy plus usual care	Usual care	NR	NR	NR	NR	HAMD, SAS, SDS, clinical effects^e^	Jadad scale
Zhang, 2015	China	SR + MA	Inception to January, 2014	10 (1092)	NR	NR	Acupuncture plus usual care	Usual care, or sham acupuncture plus usual care	NR	NR	NR	NR	CCEs, clinical effects^d^, adverse events^b^	Recommended by Cochrane Library
Chen, 2012	China	SR + MA	Inception to August, 2011	2 (206)	NR	NR	Acupuncture	Usual care	NR	NR	6 weeks or 30 sessions	NR	Clinical effects^f^and adverse events^b^	Recommended by Cochrane library
				12 (873)	NR	NR	Acupuncture plus usual care	Usual care	NR	NR	10 days to 8 weeks, or 24 sessions	NR	CCEs^g^, clinical effects^f^,and adverse events^b^	
Nery, 2014	Brazil	SR	Inception to July, 2012	1 (20)	68(4)	35	Tai Chi Chuan	Physical activity counseling	NR	NR	12 months	NR	VO_2_peak	Recommended by Cochrane Library

Abbreviations: 6MWT, 6-minute walking test; CCEs, composite cardiovascular events; ECG, electrocardiograph; HAMD, Hamilton Depression Scale; MA, meta-analysis; METs, metabolic equivalents; MLHFQ, Minnesota Living with Heart Failure Questionnaire; NR, not reported; RCTs, randomized controlled trials; RoB, risk of bias; SAQ, Seattle Angina Questionnaire; SAS, Self-Rating Anxiety Scale; SD, standard deviation; SDS, Self-Rating Depression Scale; SF-36, 36-Item Short Form Health Survey; SR, systematic review; TCM, traditional Chinese medicine; VO_2_peak, peak oxygen consumption; VO_2_/HR, oxygen consumption/heart rate. ^a^improvements in angina symptoms. ^b^adverse events related to the intervention. ^c^total clinical efficiency, improvements in symptoms and ECG. ^d^improvements in symptoms and ECG. ^e^total clinical efficiency. ^f^improvements in angina symptoms and ECG, time to onset of angina relief in response to treatment. ^g^non-fatal myocardial infarction.

**Table 2:** Summary of evidence across outcomes from included reviews.

Intervention and comparator	Number of primary studies	Type of publication	Findings^a^ and certainty of evidence (GRADE)			
			High^b^	Moderate^c^	Low^d^	Very low^e^
*Outcome: composite cardiovascular events (CCEs)*						
Acupuncture plus usual care vs usual care	3	SR + MA			+1	
		SR				+1
TCM exercise therapies plus usual care vs usual care	1	SR			+1	

*Outcome: clinical effects (total clinical efficiency: improvements in symptoms and ECG)*						
Acupuncture plus usual care vs usual care	2	SR + MA			+1	
TCM emotional therapy plus usual care vs usual care	7	SR + MA			+1	

*Outcome: clinical effects (improvements in symptoms)*						
Acupuncture plus usual care vs usual care	21	SR + MA		+3		
		SR				+1
Acupuncture vs usual care	2	SR + MA		+1		
TCM exercise therapies plus usual care vs usual care	2	SR + MA		+1		

*Outcome: clinical effects (improvements in ECG)*						
Acupuncture plus usual care vs usual care	17	SR + MA		+3		
		SR				−1
Acupuncture vs usual care	2	SR + MA		+1		

*Outcome: clinical effects (time to onset of angina relief in response to treatment)*						
Acupuncture plus usual care vs usual care	2	SR + MA			+1	
Acupuncture vs usual care	2	SR + MA			−1	
Outcome: SAQ (total score)						
Acupuncture plus usual care vs usual care	2	SR				+1
Outcome: SAQ (physical limitation)						
Baduanjin plus usual care vs usual care	8	SR + MA		+1	+1	
Baduanjin vs usual medical exercise	1	SR + MA				+2
TCM exercise therapies plus usual care vs usual care	4	SR + MA		+1		

*Outcome: SAQ (angina frequency)*						
Baduanjin plus usual care vs usual care	9	SR + MA		+1	+1	
Baduanjin vs usual medical exercise	1	SR + MA				−2
TCM exercise therapies plus usual care vs usual care	4	SR + MA		+1		

*Outcome: SAQ (angina stability)*						
Baduanjin plus usual care vs usual care	9	SR + MA		+2		
Baduanjin vs usual medical exercise	1	SR + MA				−2
TCM exercise therapies plus usual care vs usual care	4	SR + MA		+1		

*Outcome: SAQ (treatment satisfaction)*						
Baduanjin plus usual care vs usual care	9	SR + MA		±1		
Baduanjin vs usual medical exercise	1	SR + MA				+2
TCM exercise therapies plus usual care vs usual care	4	SR + MA			+1	

*Outcome: SAQ (disease perception)*						
Baduanjin plus usual care vs usual care	9	SR + MA		+2		
Baduanjin vs usual medical exercise	1	SR + MA				−2
TCM exercise therapies plus usual care vs usual care	4	SR + MA			−1	
Outcome: SF-36						
Taiji plus jogging vs jogging	1	SR + MA				+1
Taiji plus usual care vs usual care	1	SR + MA				+1

*Outcome: MLHFQ*						
Taiji plus usual care vs usual care	1	SR + MA				+1

*Outcome: 6MWT*						
Baduanjin plus usual care vs usual care	5	SR + MA			+2	
Acupuncture plus usual care vs usual care	1	SR				−1

*Outcome: METs*						
Baduanjin plus usual care vs usual care	3	SR + MA			+1	

*Outcome: VO*_*2*_ *peak*						
Tai Chi Chuan vs physical activity counseling	1	SR			−1	
TCM exercise therapies plus usual care vs usual care	2	SR + MA				−1

*Outcome: VO*_*2*_ */HR*						
TCM exercise therapies plus usual care vs usual care	2	SR + MA			+1	

*Outcome: SAS*						
Acupuncture plus usual care vs usual care	1	SR				−1
Baduanjin plus usual care vs usual care	8	SR + MA		+2	+1	
TCM exercise therapies plus usual care vs usual care	3	SR + MA				+1
TCM emotional therapy plus usual care vs usual care	3	SR + MA			+1	

*Outcome: SDS*						
Acupuncture plus usual care vs usual care	1	SR				−1
Baduanjin plus usual care vs usual care	6	SR + MA		+1	+2	
TCM exercise therapies plus usual care vs usual care	3	SR + MA				+1
TCM emotional therapy plus usual care vs usual care	3	SR + MA				+1

*Outcome: HAMD*						
TCM emotional therapy plus usual care vs usual care	4	SR + MA		+1		

*Outcome: adverse events related to the intervention*						
Baduanjin plus usual care vs usual care	13	SR		−1		
Baduanjin vs usual medical exercise	1	SR			−1	
Acupuncture plus usual care vs usual care	19	SR		−2		
Acupuncture vs usual care	2	SR		−1		

Abbreviations: SR, systematic review; SR + MA, systematic review and meta-analysis; GRADE, Grading of Recommendations Assessment, Development and Evaluation. ^a^The number indicates how many SR + MAs or SRs contributed to that result. ^b^Reviews that received no downgrades. ^c^Reviews that received 1 or 2 downgrades. ^d^Reviews that received 3 or 4 downgrades. ^e^Reviews that received 5 or 6 downgrades. + signifies statistically significant improvement in effectiveness outcomes or significantly increased risk of harms for safety outcomes. − signifies non-statistically significant change. ± signifies mixed or discordant results within SR + MAs and SRs.

## Data Availability

The data are available from the corresponding author on reasonable request.

## Abbreviations

6MWT: 6-minute walking test
AMSTAR: Assessing the Methodological Quality of Systematic Reviews tool
CCEs: Composite cardiovascular events
CHD: Chronic heart disease
ECG: Electrocardiograph
GRADE: Grading of Recommendations Assessment, Development, and Evaluation
HAMD: Hamilton Depression Scale
MA: Meta-analysis
METs: Metabolic equivalents
MLHFQ: Minnesota Living with Heart Failure Questionnaire
NPTCM: Non-pharmacological therapies of traditional Chinese medicine
PRISMA: Preferred Reporting Items for Systematic Reviews and Meta-Analyses
RCTs: Randomized clinical trials
SAQ: Seattle Angina Questionnaire
SAS: Self-Rating Anxiety Scale
SDS: Self-Rating Depression Scale
SF-36: 36-Item Short Form Health Survey
TCM: Traditional Chinese medicine
VO_2_peak: Peak oxygen consumption
VO_2_/HR: Oxygen consumption/heart rate.

## Supplementary Materials

Supplementary Materials Supplementary Material 1: PRIO-harms checklist for this overview. Supplementary Material 2: the appendices include all supplemental data and information. Appendix A: Search Strategy for Database and Gray Literature. Appendix B: PRISMA Results. Appendix C: AMSTAR Results. Appendix D: Detailed Tables of Outcome Results. Appendix E: Tables with the overlap in the primary studies included in relevant reviews. Supplementary Material 3: The matrix of evidence about primary studies across all included reviews.

## References

[1] GBD 2017 Causes of Death Collaborators (2018). Global, regional, and national age-sex-specific mortality for 282 causes of death in 195 countries and territories, 1980–2017: a systematic analysis for the global burden of disease study 2017. *LANCET*.

[2] The Writing Committee of the Report on Cardiovascular Health and Diseases in China (2021). The writing committee of the report on cardiovascular health and diseases in China: report on cardiovascular health and diseases burden in China: an updated summary of 2020. *Chinese Circulation Journal*.

[3] Liuji L., Jilan Y. (2006). Summary of ancient and modern literature on Juexintong. *Jilin Journal of Chinese Medicine*.

[4] China Association of Chinese Medicine Cardiovascular Disease Branch (2019). Guidelines for TCM diagnosis and treatment of stable angina pectoris of coronary heart disease. *Journal of Traditional Chinese Medicine*.

[5] Dejian Z., Lixing W., Zhilun Z. (2019). A summary of study on Chinese medicine characteristic therapies for coronary heart disease. *Journal of New Chinese Medicine*.

[6] Bougioukas K. I., Liakos A., Tsapas A., Ntzani E., Haidich A.-B. (2018). Preferred reporting items for overviews of systematic reviews including harms checklist: a pilot tool to be used for balanced reporting of benefits and harms. *Journal of Clinical Epidemiology*.

[7] Shea B. J., Reeves B. C., Wells G. (2017). AMSTAR 2: a critical appraisal tool for systematic reviews that include randomised or non-randomised studies of healthcare interventions, or both. *BMJ*.

[8] Page M. J., McKenzie J. E., Bossuyt P. M. (2021). The PRISMA 2020 statement: an updated guideline for reporting systematic reviews. *Journal of Clinical Epidemiology*.

[9] Pollock A., Farmer S. E., Brady M. C. (2016). An algorithm was developed to assign GRADE levels of evidence to comparisons within systematic reviews. *Journal of Clinical Epidemiology*.

[10] Pieper D., Antoine S.-L., Mathes T., Neugebauer E. A. M., Eikermann M. (2014). Systematic review finds overlapping reviews were not mentioned in every other overview. *Journal of Clinical Epidemiology*.

[11] Wenjun X., Hui T., Xiaoyun X. (2021). Effects of baduanjin on cardiac rehabilitation in patients with coronary heart disease: a meta-analysis. *Journal of Clinical Nursing*.

[12] Ling X., Yuqing Y., Yan C. (2020). Meta-analysis of the effect of baduanjin exercise on patients with coronary heart disease after PCI. *Guiding Journal of Traditional Chinese Medicine and Pharmacology*.

[13] Zhang J., Lyu S., Wu Y. (2020). Meta analysis on the efficacy and safety of exercise therapy of traditional Chinese medicine in treating stable angina pectoris of coronary heart disease. *Journal of Basic Chinese Medicine*.

[14] Naibo L. (2020). Effect of Baduanjin on quality of life of patients with coronary heart disease: a meta-analysis.

[15] Xiaoli Y., Yimin C., Xianlin W., Xiaoya J., Ren H., Wenyao X. (2019). Meta-analysis of the effect of ba duan jin on cardiac rehabilitation intervention in patients with coronary heart disease. *Medical Information*.

[16] Jianhua Z., Wei H., Jie D., Yihan T. (2018). Meta-analysis of the therapeutic effect of acupuncture on stable angina pectoris. *Journal of Hubei University of Chinese Medicine*.

[17] Zi C., Huidan Y., Ningning Z. (2018). Effect of tai chi exercise on the physiological indexes and quality of life of patients with coronary heart disease: a systematic review. *Nursing Journal of Chinese People’s Laberation Army*.

[18] Heng L., Ning X., Minglu S., Yang L., Qiwen T. (2017). Systemic review on acupuncture combined with routine treatment for unstable angenia pectoris. *Journal of Basic Chinese Medicine*.

[19] Zhang Z., Bai R., Zhang L. (2015). Acupuncture combined with western medicine for angina of coronary artery disease: a systematic review. *Chinese Acupuncture & Moxibustion*.

[20] Chen J., Ren Y., Tang Y., Li Z., Liang F. (2012). Acupuncture therapy for angina pectoris: a systematic review. *Journal of Traditional Chinese Medicine*.

[21] Tian L., Xiangying S., Yaqin W., Xiaojie M. (2016). The effects of traditional Chinese emotion nursing combined with normal nursing on patients with coronary artery disease: a systematic review. *Chinese Journal of Practical Nursing*.

[22] Nery R. M., Zanini M., Ferrari J. N. (2014). Tai chi chuan for cardiac rehabilitation in patients with coronary arterial disease. *Arquivos Brasileiros de Cardiologia*.

[23] Xiaoli L., Jingwei C., Guangqing Z., Jingying Z., Chong T. (2012). Effects of eight sections brocade (ba duan jin) on quality of life for patients after coronary artery bypass grafting. *Journal of Nursing (China)*.

[24] Knuuti J., Wijns W., Saraste A., European Society of Cardiology (ESC) (2020). 2019 ESC guidelines for the diagnosis and management of chronic coronary syndromes. *European Heart Journal*.

[25] Hongzhou G., Rongchong H. (2019). Interpretation of 2019 ESC guidelines for the diagnosis and management of chronic coronary syndromes. *Chinese Circulation Journal*.

[26] Chen X., Marrone G., Olson T. P. (2020). Intensity level and cardiorespiratory responses to baduanjin exercise in patients with chronic heart failure. *ESC Heart Failure*.

[27] Zou L., Sasaki J. E., Wang H., Xiao Z., Fang Q., Zhang M. (2017). A systematic review and meta-analysis baduanjin qigong for health benefits: randomized controlled trials. *Evidence-Based Complementary and Alternative Medicine: eCAM*.

[28] Chinese Integrative Medicine Association Cardiology Committee Psychocardiology Group (2017). Expert consensus on the diagnosis and treatment of psycho—cardiological disease with integrated traditional Chinese and western medicine. *Chinese General Practice*.

[29] Olex S., Newberg A., Figueredo V. M. (2013). Meditation: should a cardiologist care?. *International Journal of Cardiology*.

