# Supplementary material for: Effects and Safety of Non-Pharmacological Therapies of Traditional Chinese Medicine for Coronary Heart Disease: An Overview of Systematic Reviews
Source: Evid Based Complement Alternat Med. 2022 Mar 19;2022:8465269. doi: 10.1155/2022/8465269 (PMC8957469; doi:10.1155/2022/8465269)
Supplement: Supplementary Materials — Supplementary Material 1: PRIO-harms checklist for this overview. Supplementary Material 2: the appendices include all supplemental data and information. Appendix A: Search Strategy for Database and Gray Literature. Appendix B: PRISMA Results. Appendix C: AMSTAR Results. Appendix D: Detailed Tables of Outcome Results. Appendix E: Tables with the overlap in the primary studies included in relevant reviews. Supplementary Material 3: The matrix of evidence about primary studies across all included reviews. [file 8465269.f1.zip › 8465269.f1/Additiional file 2 (1).pdf]

Additional File 2

Appendix A: Search Strategy for Database and Gray Literature..2

Appendix B: PRISMA results.. 14

Appendix C: AMSTAR2 results.. 17

Appendix D: Detailed Tables of Outcome Results...18

Appendix E: Tables with the overlap in the primary studies included in relevant reviews 23

References..25

Appendix A: Search Strategy for Database and Gray Literature

Appendix A1: Search Strategy for PubMed

Search number	Query	Filters	Results
1	"Coronary Disease"[Mesh]		222,088
2	(coronary heart disease*[Title/Abstract]) OR (coronary disease*[Title/Abstract])		70,918
3	"Angina Pectoris"[Mesh]		43,774
4	(stenocardia*[Title/Abstract]) OR (angor pectoris[Title/Abstract])		994
5	"Myocardial Infarction"[Mesh]		179,541
6	(((myocardial infarction*[Title/Abstract]) OR (cardiovascular stroke*[Title/Abstract])) OR (myocardial infarct*[Title/Abstract])) OR (heart attack*[Title/Abstract])		211,575
7	((((("Coronary Disease"[Mesh]) OR ((coronary heart disease*[Title/Abstract]) OR (coronary disease*[Title/Abstract]))) OR ("Angina Pectoris"[Mesh])) OR ((stenocardia*[Title/Abstract]) OR (angor pectoris[Title/Abstract]))) OR ("Myocardial Infarction"[Mesh])) OR ((((myocardial infarction*[Title/Abstract]) OR (cardiovascular stroke*[Title/Abstract])) OR (myocardial infarct*[Title/Abstract])) OR (heart attack*[Title/Abstract]))		482,500
8	"Acupuncture Therapy"[Mesh]		25,678
9	(acupuncture treatment*[Title/Abstract]) OR (acupotom*[Title/Abstract])		3,159
10	"Acupuncture, Ear"[Mesh]		453
11	(ear acupuncture*[Title/Abstract]) OR (auricular acupuncture*[Title/Abstract])		556
12	"Electroacupuncture"[Mesh]		4,201
13	Electroacupuncture[Title/Abstract]		5,589
14	"Moxibustion"[Mesh]		2,248
15	Moxibustion[Title/Abstract]		2,917
16	"Massage"[Mesh]		6,328
17	(((Zone Therap*[Title/Abstract]) OR (Massage Therap*[Title/Abstract])) OR (tuina[Title/Abstract])) OR (tui-na[Title/Abstract])		1,700
18	"Cupping Therapy"[Mesh]		41
19	(Cupping Treatment*[Title/Abstract]) OR (Cupping Therap*[Title/Abstract])		265
20	(scrapping therap*[Title/Abstract]) OR (scraping therap*[Title/Abstract])		26
21	"Tai Ji"[Mesh]		1,192
22	((((((Tai-ji[Title/Abstract]) OR (Tai Chi[Title/Abstract])) OR (Tai Ji Quan[Title/Abstract])) OR (Taiji[Title/Abstract])) OR (Taijiquan[Title/Abstract])) OR (T'ai Chi[Title/Abstract])) OR (Tai Chi Chuan[Title/Abstract])		2,061
23	"Qigong"[Mesh]		234
24	(Qi Gong[Title/Abstract]) OR (Ch'i Kung[Title/Abstract])		115

25	("baduanjin"[Title/Abstract]) OR ("eight-section* exercise"[Title/Abstract])		118
26	"Diet Therapy"[Mesh]		56,622
27	(((((Diet Therap*[Title/Abstract]) OR (Restrictive Diet Therap*[Title/Abstract])) OR (Restriction Diet Therap*[Title/Abstract])) OR (Dietary Restriction*[Title/Abstract])) OR (Dietary Modification*[Title/Abstract])) OR (Diet Modification*[Title/Abstract])		12,338
28	"Psychotherapy"[Mesh]		201,495
29	Psychotherap*[Title/Abstract]		48,567
30	"Music Therapy"[Mesh]		3,725
31	Music Therap*[Title/Abstract]		2,720
32	(((((((((((((((((((((("Acupuncture Therapy"[Mesh]) OR ((acupuncture treatment*[Title/Abstract]) OR (acupotom*[Title/Abstract]))) OR ("Acupuncture, Ear"[Mesh])) OR ((ear acupuncture*[Title/Abstract]) OR (auricular acupuncture*[Title/Abstract]))) OR ("Electroacupuncture"[Mesh])) OR (Electroacupuncture[Title/Abstract])) OR ("Moxibustion"[Mesh])) OR (Moxibustion[Title/Abstract])) OR ("Massage"[Mesh])) OR ((((Zone Therap*[Title/Abstract]) OR (Massage Therap*[Title/Abstract])) OR (tuina[Title/Abstract])) OR (tui-na[Title/Abstract]))) OR ("Cupping Therapy"[Mesh])) OR ((Cupping Treatment*[Title/Abstract]) OR (Cupping Therap*[Title/Abstract]))) OR ((scrapping therap*[Title/Abstract]) OR (scraping therap*[Title/Abstract]))) OR ("Tai Ji"[Mesh])) OR (((((((Tai-ji[Title/Abstract]) OR (Tai Chi[Title/Abstract])) OR (Tai Ji Quan[Title/Abstract])) OR (Taiji[Title/Abstract])) OR (Taijiquan[Title/Abstract])) OR (T'ai Chi[Title/Abstract])) OR (Tai Chi Chuan[Title/Abstract]))) OR ("Qigong"[Mesh])) OR ((Qi Gong[Title/Abstract]) OR (Ch'i Kung[Title/Abstract]))) OR (("baduanjin"[Title/Abstract]) OR ("eight-section* exercise"[Title/Abstract]))) OR ("Diet Therapy"[Mesh])) OR ((((((Diet Therap*[Title/Abstract]) OR (Restrictive Diet Therap*[Title/Abstract])) OR (Restriction Diet Therap*[Title/Abstract])) OR (Dietary Restriction*[Title/Abstract])) OR (Dietary Modification*[Title/Abstract])) OR (Diet Modification*[Title/Abstract]))) OR ("Psychotherapy"[Mesh])) OR (Psychotherap*[Title/Abstract])) OR ("Music Therapy"[Mesh])) OR (Music Therap*[Title/Abstract])		316,172
33	((((("Coronary Disease"[Mesh]) OR ((coronary heart disease*[Title/Abstract]) OR (coronary disease*[Title/Abstract]))) OR ("Angina Pectoris"[Mesh])) OR ((stenocardia*[Title/Abstract]) OR (angor pectoris[Title/Abstract]))) OR ("Myocardial Infarction"[Mesh])) OR ((((myocardial infarction*[Title/Abstract]) OR (cardiovascular stroke*[Title/Abstract])) OR (myocardial infarct*[Title/Abstract])) OR (heart attack*[Title/Abstract]))) AND (((((((((((((((((((((("Acupuncture Therapy"[Mesh]) OR ((acupuncture treatment*[Title/Abstract]) OR (acupotom*[Title/Abstract]))) OR ("Acupuncture, Ear"[Mesh])) OR ((ear acupuncture*[Title/Abstract]) OR (auricular acupuncture*[Title/Abstract]))) OR ("Electroacupuncture"[Mesh])) OR (Electroacupuncture[Title/Abstract])) OR ("Moxibustion"[Mesh])) OR (Moxibustion[Title/Abstract])) OR ("Massage"[Mesh])) OR ((((Zone Therap*[Title/Abstract]) OR (Massage Therap*[Title/Abstract])) OR (tuina[Title/Abstract])) OR (tui-na[Title/Abstract]))) OR ("Cupping Therapy"[Mesh])) OR ((Cupping Treatment*[Title/Abstract]) OR (Cupping Therap*[Title/Abstract]))) OR ((scrapping therap*[Title/Abstract]) OR (scraping therap*[Title/Abstract]))) OR ("Tai Ji"[Mesh])) OR (((((((Tai-ji[Title/Abstract]) OR (Tai Chi[Title/Abstract])) OR (Tai Ji Quan[Title/Abstract])) OR (Taiji[Title/Abstract])) OR (Taijiquan[Title/Abstract])) OR (T'ai Chi[Title/Abstract])) OR (Tai Chi Chuan[Title/Abstract]))) OR ("Qigong"[Mesh])) OR ((Qi Gong[Title/Abstract]) OR (Ch'i Kung[Title/Abstract]))) OR (("baduanjin"[Title/Abstract]) OR ("eight-section* exercise"[Title/Abstract]))) OR ("Diet Therapy"[Mesh])) OR ((((((Diet Therap*[Title/Abstract]) OR (Restrictive Diet Therap*[Title/Abstract])) OR (Restriction Diet Therap*[Title/Abstract])) OR (Dietary Restriction*[Title/Abstract])) OR (Dietary Modification*[Title/Abstract])) OR (Diet Modification*[Title/Abstract]))) OR ("Psychotherapy"[Mesh])) OR (Psychotherap*[Title/Abstract])) OR ("Music Therapy"[Mesh])) OR (Music Therap*[Title/Abstract]))		3,689
34	((((("Coronary Disease"[Mesh]) OR ((coronary heart disease*[Title/Abstract]) OR (coronary disease*[Title/Abstract]))) OR ("Angina Pectoris"[Mesh])) OR ((stenocardia*[Title/Abstract]) OR (angor pectoris[Title/Abstract]))) OR ("Myocardial Infarction"[Mesh])) OR ((((myocardial infarction*[Title/Abstract]) OR (cardiovascular stroke*[Title/Abstract])) OR (myocardial	Systematic Review	96

	infarct*[Title/Abstract])) OR (heart attack*[Title/Abstract]))) AND ((((((((((((((((((((((("Acupuncture Therapy"[Mesh]) OR ((acupuncture treatment*[Title/Abstract]) OR (acupotom*[Title/Abstract]))) OR ("Acupuncture, Ear"[Mesh])) OR ((ear acupuncture*[Title/Abstract]) OR (auricular acupuncture*[Title/Abstract]))) OR ("Electroacupuncture"[Mesh])) OR (Electroacupuncture[Title/Abstract])) OR ("Moxibustion"[Mesh])) OR (Moxibustion[Title/Abstract])) OR ("Massage"[Mesh])) OR ((((Zone Therap*[Title/Abstract]) OR (Massage Therap*[Title/Abstract])) OR (tuina[Title/Abstract])) OR (tui-na[Title/Abstract]))) OR ("Cupping Therapy"[Mesh])) OR ((Cupping Treatment*[Title/Abstract]) OR (Cupping Therap*[Title/Abstract]))) OR ((scrapping therap*[Title/Abstract]) OR (scraping therap*[Title/Abstract]))) OR ("Tai Ji"[Mesh])) OR (((((((Tai-ji[Title/Abstract]) OR (Tai Chi[Title/Abstract])) OR (Tai Ji Quan[Title/Abstract])) OR (Taiji[Title/Abstract])) OR (Taijiquan[Title/Abstract])) OR (T'ai Chi[Title/Abstract])) OR (Tai Chi Chuan[Title/Abstract]))) OR ("Qigong"[Mesh])) OR ((Qi Gong[Title/Abstract]) OR (Ch'i Kung[Title/Abstract]))) OR (("baduanjin"[Title/Abstract]) OR ("eight-section* exercise"[Title/Abstract]))) OR ("Diet Therapy"[Mesh])) OR ((((((Diet Therap*[Title/Abstract]) OR (Restrictive Diet Therap*[Title/Abstract])) OR (Restriction Diet Therap*[Title/Abstract])) OR (Dietary Restriction*[Title/Abstract])) OR (Dietary Modification*[Title/Abstract])) OR (Diet Modification*[Title/Abstract]))) OR ("Psychotherapy"[Mesh])) OR (Psychotherap*[Title/Abstract])) OR ("Music Therapy"[Mesh])) OR (Music Therap*[Title/Abstract]))		
35	(((((("Coronary Disease"[Mesh]) OR ((coronary heart disease*[Title/Abstract]) OR (coronary disease*[Title/Abstract]))) OR ("Angina Pectoris"[Mesh])) OR ((stenocardia*[Title/Abstract]) OR (angor pectoris[Title/Abstract]))) OR ("Myocardial Infarction"[Mesh])) OR ((((myocardial infarction*[Title/Abstract]) OR (cardiovascular stroke*[Title/Abstract])) OR (myocardial infarct*[Title/Abstract])) OR (heart attack*[Title/Abstract]))) AND ((((((((((((((((((((((("Acupuncture Therapy"[Mesh]) OR ((acupuncture treatment*[Title/Abstract]) OR (acupotom*[Title/Abstract]))) OR ("Acupuncture, Ear"[Mesh])) OR ((ear acupuncture*[Title/Abstract]) OR (auricular acupuncture*[Title/Abstract]))) OR ("Electroacupuncture"[Mesh])) OR (Electroacupuncture[Title/Abstract])) OR ("Moxibustion"[Mesh])) OR (Moxibustion[Title/Abstract])) OR ("Massage"[Mesh])) OR ((((Zone Therap*[Title/Abstract]) OR (Massage Therap*[Title/Abstract])) OR (tuina[Title/Abstract])) OR (tui-na[Title/Abstract]))) OR ("Cupping Therapy"[Mesh])) OR ((Cupping Treatment*[Title/Abstract]) OR (Cupping Therap*[Title/Abstract]))) OR ((scrapping therap*[Title/Abstract]) OR (scraping therap*[Title/Abstract]))) OR ("Tai Ji"[Mesh])) OR (((((((Tai-ji[Title/Abstract]) OR (Tai Chi[Title/Abstract])) OR (Tai Ji Quan[Title/Abstract])) OR (Taiji[Title/Abstract])) OR (Taijiquan[Title/Abstract])) OR (T'ai Chi[Title/Abstract])) OR (Tai Chi Chuan[Title/Abstract]))) OR ("Qigong"[Mesh])) OR ((Qi Gong[Title/Abstract]) OR (Ch'i Kung[Title/Abstract]))) OR (("baduanjin"[Title/Abstract]) OR ("eight-section* exercise"[Title/Abstract]))) OR ("Diet Therapy"[Mesh])) OR ((((((Diet Therap*[Title/Abstract]) OR (Restrictive Diet Therap*[Title/Abstract])) OR (Restriction Diet Therap*[Title/Abstract])) OR (Dietary Restriction*[Title/Abstract])) OR (Dietary Modification*[Title/Abstract])) OR (Diet Modification*[Title/Abstract]))) OR ("Psychotherapy"[Mesh])) OR (Psychotherap*[Title/Abstract])) OR ("Music Therapy"[Mesh])) OR (Music Therap*[Title/Abstract]))	Meta-Analysis, Systematic Review	128
36	(((((("Coronary Disease"[Mesh]) OR ((coronary heart disease*[Title/Abstract]) OR (coronary disease*[Title/Abstract]))) OR ("Angina Pectoris"[Mesh])) OR ((stenocardia*[Title/Abstract]) OR (angor pectoris[Title/Abstract]))) OR ("Myocardial Infarction"[Mesh])) OR ((((myocardial infarction*[Title/Abstract]) OR (cardiovascular stroke*[Title/Abstract])) OR (myocardial infarct*[Title/Abstract])) OR (heart attack*[Title/Abstract]))) AND ((((((((((((((((((((((("Acupuncture Therapy"[Mesh]) OR ((acupuncture treatment*[Title/Abstract]) OR (acupotom*[Title/Abstract]))) OR ("Acupuncture, Ear"[Mesh])) OR ((ear acupuncture*[Title/Abstract]) OR (auricular acupuncture*[Title/Abstract]))) OR ("Electroacupuncture"[Mesh])) OR (Electroacupuncture[Title/Abstract])) OR ("Moxibustion"[Mesh])) OR (Moxibustion[Title/Abstract])) OR ("Massage"[Mesh])) OR ((((Zone Therap*[Title/Abstract]) OR (Massage Therap*[Title/Abstract])) OR (tuina[Title/Abstract])) OR (tui-na[Title/Abstract]))) OR ("Cupping Therapy"[Mesh])) OR ((Cupping Treatment*[Title/Abstract]) OR (Cupping Therap*[Title/Abstract]))) OR ((scrapping therap*[Title/Abstract]) OR (scraping therap*[Title/Abstract]))) OR ("Tai Ji"[Mesh])) OR (((((((Tai-ji[Title/Abstract]) OR (Tai Chi[Title/Abstract])) OR (Tai Ji Quan[Title/Abstract])) OR (Taiji[Title/Abstract])) OR (Taijiquan[Title/Abstract])) OR (T'ai Chi[Title/Abstract])) OR (Tai Chi Chuan[Title/Abstract]))) OR ("Qigong"[Mesh])) OR ((Qi Gong[Title/Abstract]) OR (Ch'i Kung[Title/Abstract]))) OR (("baduanjin"[Title/Abstract]) OR ("eight-section* exercise"[Title/Abstract]))) OR ("Diet Therapy"[Mesh])) OR ((((((Diet	Meta-Analysis, Systematic Review, Humans	114

	Therap*[Title/Abstract]) OR (Restrictive Diet Therap*[Title/Abstract])) OR (Restriction Diet Therap*[Title/Abstract])) OR (Dietary Restriction*[Title/Abstract])) OR (Dietary Modification*[Title/Abstract])) OR (Diet Modification*[Title/Abstract]))) OR ("Psychotherapy"[Mesh])) OR (Psychotherap*[Title/Abstract])) OR ("Music Therapy"[Mesh])) OR (Music Therap*[Title/Abstract]))

Appendix A2: Search Strategy for Cochrane Library

ID	Search
#1	MeSH descriptor: [Coronary Disease] explode all trees
#2	(Coronary Diseases):ti,ab,kw OR (coronary heart disease*):ti,ab,kw OR (disease*, coronary):ti,ab,kw OR (disease*, coronary Heart):ti,ab,kw OR (heart disease*, coronary):ti,ab,kw
#3	MeSH descriptor: [Angina Pectoris] explode all trees
#4	("angor pectoris"):ti,ab,kw OR (stenocardia*):ti,ab,kw
#5	MeSH descriptor: [Myocardial Infarction] explode all trees
#6	(infarct*, myocardial):ti,ab,kw OR (stroke*, cardiovascular):ti,ab,kw OR (cardiovascular stroke*):ti,ab,kw OR (myocardial infarct*):ti,ab,kw OR (heart attack*):ti,ab,kw
#7	#1 OR #2 OR #3 OR #4 OR #5 OR #6
#8	MeSH descriptor: [Acupuncture Therapy] explode all trees
#9	(treatment*, acupuncture):ti,ab,kw OR (acupuncture treatment*):ti,ab,kw OR (therap*, acupuncture):ti,ab,kw OR (acupotom*):ti,ab,kw
#10	MeSH descriptor: [Acupuncture, Ear] explode all trees
#11	(ear acupuncture*):ti,ab,kw OR (acupuncture*, auricular):ti,ab,kw OR (auricular acupuncture*):ti,ab,kw OR (acupuncture*, ear):ti,ab,kw
#12	MeSH descriptor: [Electroacupuncture] explode all trees
#13	(electroacupuncture):ti,ab,kw
#14	MeSH descriptor: [Moxibustion] explode all trees
#15	(moxabustion):ti,ab,kw
#16	MeSH descriptor: [Massage] explode all trees
#17	(therap*, zone):ti,ab,kw OR (zone therap*):ti,ab,kw OR (therap*, massage):ti,ab,kw OR (massage therap*):ti,ab,kw OR (tuina):ti,ab,kw
#18	MeSH descriptor: [Cupping Therapy] explode all trees
#19	(cupping treatment*):ti,ab,kw OR (therapy, cupping):ti,ab,kw OR (cupping therap*):ti,ab,kw OR (treatment, cupping):ti,ab,kw
#20	(scrapping):ti,ab,kw OR (scraping):ti,ab,kw
#21	MeSH descriptor: [Tai Ji] explode all trees
#22	(Taiji*):ti,ab,kw OR (T?ai Chi):ti,ab,kw OR (Ji Quan, Tai):ti,ab,kw OR ("Tai Ji Quan"):ti,ab,kw OR (Quan, Tai Ji):ti,ab,kw

#23	MeSH descriptor: [Qigong] explode all trees
#24	(Qi Gong):ti,ab,kw OR (Ch'i Kung):ti,ab,kw
#25	(Baduanjin):ti,ab,kw OR (Baduanjin exercise):ti,ab,kw OR (eight-section* exercise):ti,ab,kw
#26	MeSH descriptor: [Diet Therapy] explode all trees
#27	(diet* modification*):ti,ab,kw OR (modification, diet*):ti,ab,kw OR (diet therap*):ti,ab,kw OR (therapy, diet):ti,ab,kw
#28	MeSH descriptor: [Psychotherapy] explode all trees
#29	(Psychotherap*):ti,ab,kw
#30	MeSH descriptor: [Music Therapy] explode all trees
#31	(Therapy, Music):ti,ab,kw
#32	{OR #8-#31}
#33	#7 AND #32 in Cochrane Reviews

Appendix A3: Search Strategy for Embase

No.	Query
#1	ischemic heart disease'/exp
#2	'coronary artery insufficiency':ab,ti OR 'coronary artery occlusive disease':ab,ti OR 'coronary heart disease':ab,ti OR 'coronary insufficiency':ab,ti OR 'coronary occlusive disease':ab,ti OR 'heart disease, coronary':ab,ti OR 'heart disease, ischaemic':ab,ti OR 'heart disease, ischemic':ab,ti OR 'ischaemia heart disease':ab,ti OR 'ischaemic cardiac disease':ab,ti OR 'ischaemic cardial disease':ab,ti OR 'ischaemic cardiopathy':ab,ti OR 'ischaemic heart disease':ab,ti OR 'ischemia heart disease':ab,ti OR 'ischemic cardiac disease':ab,ti OR 'ischemic cardial disease':ab,ti OR 'ischemic cardiopathy':ab,ti
#3	'heart infarction'/exp
#4	'cardiac infarct':ab,ti OR 'cardiac infarction':ab,ti OR 'cardial infarct':ab,ti OR 'heart attack':ab,ti OR 'heart infarct':ab,ti OR 'heart micro infarction':ab,ti OR 'heart muscle infarction':ab,ti OR 'infarction, heart':ab,ti OR 'myocardial infarct':ab,ti OR 'myocardial infarction':ab,ti OR 'myocardium infarct':ab,ti OR 'myocardium infarction':ab,ti OR 'premonitory infarction sign':ab,ti OR 'second heart attack':ab,ti OR 'subendocardial infarction':ab,ti OR 'transmural cardiac infarction':ab,ti OR 'transmural heart infarction':ab,ti OR 'transmural infarction, heart':ab,ti
#5	'angina pectoris'/exp
#6	'angina':ab,ti OR 'anginal attack':ab,ti OR 'effort angina pectoris':ab,ti OR 'postprandial angina pectoris':ab,ti OR 'stenocardia':ab,ti
#7	#1 OR #2 OR #3 OR #4 OR #5 OR #6
#8	'acupuncture'/exp
#9	'acupuncture therapy':ab,ti OR 'shonishin':ab,ti
#10	'moxibustion'/exp
#11	'moxibustion':ab,ti
#12	'auricular acupuncture'/exp

#13	'acupuncture, ear':ab,ti OR 'acupuncture, earlobe':ab,ti OR 'auriculo-acupuncture':ab,ti OR 'auriculoacupuncture':ab,ti OR 'auriculotherapy':ab,ti OR 'ear acupuncture':ab,ti OR 'earlobe acupuncture':ab,ti
#14	'electroacupuncture'/exp
#15	'acupuncture, electric':ab,ti OR 'electric acupuncture':ab,ti OR 'electrical acupoint stimulation':ab,ti OR 'electrical acupuncture':ab,ti OR 'electro-acupuncture':ab,ti OR 'electrode acupuncture':ab,ti OR 'electronic acupuncture':ab,ti
#16	'massage'/exp
#17	'massage therapy':ab,ti OR 'masso-therapy':ab,ti OR 'massotherapy':ab,ti OR 'sports massage':ab,ti
#18	'tui na'/exp
#19	'tuina'/exp
#20	'tuina':ab,ti OR 'tui na':ab,ti
#21	'cupping therapy'/exp
#22	'cupping (therapy)':ab,ti OR 'cupping manipulation':ab,ti OR 'cupping treatment':ab,ti OR 'fire cupping':ab,ti OR 'flash cupping':ab,ti OR 'moving cupping':ab,ti OR 'suction cupping':ab,ti OR 'vacuum cupping':ab,ti
#23	'scraping'/exp
#24	'scraping':ab,ti OR 'scraping therapy':ab,ti
#25	'tai chi'/exp
#26	'tai chi chuan':ab,ti OR 'tai ji':ab,ti OR 'taiji quan':ab,ti OR 'taijiquan':ab,ti
#27	'qigong'/exp
#28	'chi kung':ab,ti OR 'chigung':ab,ti OR 'qi gong':ab,ti
#29	'baduanjin'/exp
#30	'baduanjin exercise':ab,ti OR 'eight-sectioned exercise':ab,ti OR 'eight-section exercise':ab,ti
#31	'diet therapy'/exp
#32	'diet intervention':ab,ti OR 'diet treatment':ab,ti OR 'dietary intervention':ab,ti OR 'dietary therapy':ab,ti OR 'dietary treatment':ab,ti OR 'nutrition therapy':ab,ti OR 'nutritional therapy':ab,ti
#33	'psychotherapy'/exp
#34	'holistic psychotherapy':ab,ti OR 'multiple psychotherapy':ab,ti OR 'psychotherapeutic processes':ab,ti OR 'psychotherapeutic training':ab,ti OR 'psychotherapy, multiple':ab,ti OR 'socioenvironmental therapy':ab,ti
#35	'music therapy'/exp
#36	'music therapy':ab,ti OR 'therapy, music':ab,ti
#37	#8 OR #9 OR #10 OR #11 OR #12 OR #13 OR #14 OR #15 OR #16 OR #17 OR #18 OR #19 OR #20 OR #21 OR #22 OR #23 OR #24 OR #25 OR #26 OR #27 OR #28 OR #29 OR #30 OR #31 OR #32 OR #33 OR #34 OR #35 OR #36
#38	#7 AND #37
#39	#38 AND ('meta analysis'/de OR 'systematic review'/de)

Appendix A4: Search Strategy for Epistemonikos

#	Query
**1**	(title:(coronary disease*) OR abstract:(coronary disease*)) OR (title:(coronary heart disease*) OR abstract:(coronary heart disease*)) OR (title:(disease*, coronary) OR abstract:(disease*, coronary)) OR (title:(disease*, coronary heart) OR abstract:(disease*, coronary heart)) OR (title:(heart disease*, coronary) OR abstract:(heart disease*, coronary))
**2**	(title:(angina pectoris) OR abstract:(angina pectoris)) OR (title:(angor pectoris) OR abstract:(angor pectoris)) OR (title:(stenocardia*) OR abstract:(stenocardia*))
**3**	(title:(myocardial infarct*) OR abstract:(myocardial infarct*)) OR (title:(infarct*, myocardial) OR abstract:(infarct*, myocardial)) OR (title:(stroke*, cardiovascular) OR abstract:(stroke*, cardiovascular)) OR (title:(cardiovascular stroke*) OR abstract:(cardiovascular stroke*)) OR (title:(heart attack*) OR abstract:(heart attack*))
**4**	(title:(coronary disease*) OR abstract:(coronary disease*)) OR (title:(coronary heart disease*) OR abstract:(coronary heart disease*)) OR (title:(disease*, coronary) OR abstract:(disease*, coronary)) OR (title:(disease*, coronary heart) OR abstract:(disease*, coronary heart)) OR (title:(heart disease*, coronary) OR abstract:(heart disease*, coronary)) OR (title:(angina pectoris) OR abstract:(angina pectoris)) OR (title:(angor pectoris) OR abstract:(angor pectoris)) OR (title:(stenocardia*) OR abstract:(stenocardia*)) OR (title:(myocardial infarct*) OR abstract:(myocardial infarct*)) OR (title:(infarct*, myocardial) OR abstract:(infarct*, myocardial)) OR (title:(stroke*, cardiovascular) OR abstract:(stroke*, cardiovascular)) OR (title:(cardiovascular stroke*) OR abstract:(cardiovascular stroke*)) OR (title:(heart attack*) OR abstract:(heart attack*))
**5**	(title:(acupuncture therapy) OR abstract:(acupuncture therapy)) OR (title:(treatment*, acupuncture) OR abstract:(treatment*, acupuncture)) OR (title:(acupuncture treatment*) OR abstract:(acupuncture treatment*)) OR (title:(therap*, acupuncture) OR abstract:(therap*, acupuncture)) OR (title:(acupotom*) OR abstract:(acupotom*))
**6**	(title:(acupuncture*, ear) OR abstract:(acupuncture*, ear)) OR (title:(ear acupuncture*) OR abstract:(ear acupuncture*)) OR (title:(acupuncture*, auricular) OR abstract:(acupuncture*, auricular)) OR (title:(auricular acupuncture*) OR abstract:(auricular acupuncture*)) OR (title:(acupuncture*, ear) OR abstract:(acupuncture*, ear))
**7**	(title:(electroacupuncture) OR abstract:(electroacupuncture))
**8**	(title:(moxibustion) OR abstract:(moxibustion)) OR (title:(moxabustion) OR abstract:(moxabustion))
**9**	(title:(massage) OR abstract:(massage)) OR (title:(therap*, zone) OR abstract:(therap*, zone)) OR (title:(zone therap*) OR abstract:(zone therap*)) OR (title:(therap*, massage) OR abstract:(therap*, massage)) OR (title:(massage therap*) OR abstract:(massage therap*)) OR (title:(tuina) OR abstract:(tuina))
**10**	(title:(cupping therap*) OR abstract:(cupping therap*)) OR (title:(cupping treatment*) OR abstract:(cupping treatment*)) OR (title:(therapy, cupping) OR abstract:(therapy, cupping)) OR (title:(treatment, cupping) OR abstract:(treatment, cupping))
**11**	(title:(scrapping) OR abstract:(scrapping)) OR (title:(scraping) OR abstract:(scraping))
**12**	(title:(Tai Ji) OR abstract:(Tai Ji)) OR (title:(Taiji*) OR abstract:(Taiji*)) OR (title:(T?ai Chi) OR abstract:(T?ai Chi)) OR (title:(Ji Quan, Tai) OR abstract:(Ji Quan, Tai)) OR (title:(Tai Ji Quan) OR abstract:(Tai Ji Quan)) OR (title:(Quan, Tai Ji) OR abstract:(Quan, Tai Ji))
**13**	(title:(Qigong) OR abstract:(Qigong)) OR (title:(Qi Gong) OR abstract:(Qi Gong)) OR (title:(Ch'i Kung) OR abstract:(Ch'i Kung))
**14**	(title:(Baduanjin) OR abstract:(Baduanjin)) OR (title:(Baduanjin exercise) OR abstract:(Baduanjin exercise)) OR (title:(eight-section* exercise) OR abstract:(eight-section* exercise))
**15**	(title:(Diet Therapy) OR abstract:(Diet Therapy)) OR (title:(diet* modification*) OR abstract:(diet* modification*)) OR (title:(modification, diet*) OR abstract:(modification, diet*)) OR (title:(diet therap*) OR abstract:(diet therap*)) OR (title:(therapy, diet) OR abstract:(therapy, diet))
**16**	(title:(Psychotherap*) OR abstract:(Psychotherap*))

**17**	(title:(Music Therapy) OR abstract:(Music Therapy)) OR (title:(Therapy, Music) OR abstract:(Therapy, Music))
**18**	(title:(acupuncture therapy) OR abstract:(acupuncture therapy)) OR (title:(treatment*, acupuncture) OR abstract:(treatment*, acupuncture)) OR (title:(acupuncture treatment*) OR abstract:(acupuncture treatment*)) OR (title:(therap*, acupuncture) OR abstract:(therap*, acupuncture)) OR (title:(acupotom*) OR abstract:(acupotom*)) OR (title:(acupuncture*, ear) OR abstract:(acupuncture*, ear)) OR (title:(ear acupuncture*) OR abstract:(ear acupuncture*)) OR (title:(acupuncture*, auricular) OR abstract:(acupuncture*, auricular)) OR (title:(auricular acupuncture*) OR abstract:(auricular acupuncture*)) OR (title:(acupuncture*, ear) OR abstract:(acupuncture*, ear)) OR (title:(electroacupuncture) OR abstract:(electroacupuncture)) OR (title:(moxibustion) OR abstract:(moxibustion)) OR (title:(moxabustion) OR abstract:(moxabustion)) OR (title:(massage) OR abstract:(massage)) OR (title:(therap*, zone) OR abstract:(therap*, zone)) OR (title:(zone therap*) OR abstract:(zone therap*)) OR (title:(therap*, massage) OR abstract:(therap*, massage)) OR (title:(massage therap*) OR abstract:(massage therap*)) OR (title:(tuina) OR abstract:(tuina)) OR (title:(cupping therap*) OR abstract:(cupping therap*)) OR (title:(cupping treatment*) OR abstract:(cupping treatment*)) OR (title:(therapy, cupping) OR abstract:(therapy, cupping)) OR (title:(treatment, cupping) OR abstract:(treatment, cupping)) OR (title:(scrapping) OR abstract:(scrapping)) OR (title:(scraping) OR abstract:(scraping)) OR (title:(Tai Ji) OR abstract:(Tai Ji)) OR (title:(Taiji*) OR abstract:(Taiji*)) OR (title:(T?ai Chi) OR abstract:(T?ai Chi)) OR (title:(Ji Quan, Tai) OR abstract:(Ji Quan, Tai)) OR (title:(Tai Ji Quan) OR abstract:(Tai Ji Quan)) OR (title:(Quan, Tai Ji) OR abstract:(Quan, Tai Ji)) OR (title:(Qigong) OR abstract:(Qigong)) OR (title:(Qi Gong) OR abstract:(Qi Gong)) OR (title:(Ch'i Kung) OR abstract:(Ch'i Kung)) OR (title:(Baduanjin) OR abstract:(Baduanjin)) OR (title:(Baduanjin exercise) OR abstract:(Baduanjin exercise)) OR (title:(eight-section* exercise) OR abstract:(eight-section* exercise)) OR (title:(Diet Therapy) OR abstract:(Diet Therapy)) OR (title:(diet* modification*) OR abstract:(diet* modification*)) OR (title:(modification, diet*) OR abstract:(modification, diet*)) OR (title:(diet therap*) OR abstract:(diet therap*)) OR (title:(therapy, diet) OR abstract:(therapy, diet)) OR (title:(Psychotherap*) OR abstract:(Psychotherap*)) OR (title:(Music Therapy) OR abstract:(Music Therapy)) OR (title:(Therapy, Music) OR abstract:(Therapy, Music))
**19**	((title:(acupuncture therapy) OR abstract:(acupuncture therapy)) OR (title:(treatment*, acupuncture) OR abstract:(treatment*, acupuncture)) OR (title:(acupuncture treatment*) OR abstract:(acupuncture treatment*)) OR (title:(therap*, acupuncture) OR abstract:(therap*, acupuncture)) OR (title:(acupotom*) OR abstract:(acupotom*)) OR (title:(acupuncture*, ear) OR abstract:(acupuncture*, ear)) OR (title:(ear acupuncture*) OR abstract:(ear acupuncture*)) OR (title:(acupuncture*, auricular) OR abstract:(acupuncture*, auricular)) OR (title:(auricular acupuncture*) OR abstract:(auricular acupuncture*)) OR (title:(acupuncture*, ear) OR abstract:(acupuncture*, ear)) OR (title:(electroacupuncture) OR abstract:(electroacupuncture)) OR (title:(moxibustion) OR abstract:(moxibustion)) OR (title:(moxabustion) OR abstract:(moxabustion)) OR (title:(massage) OR abstract:(massage)) OR (title:(therap*, zone) OR abstract:(therap*, zone)) OR (title:(zone therap*) OR abstract:(zone therap*)) OR (title:(therap*, massage) OR abstract:(therap*, massage)) OR (title:(massage therap*) OR abstract:(massage therap*)) OR (title:(tuina) OR abstract:(tuina)) OR (title:(cupping therap*) OR abstract:(cupping therap*)) OR (title:(cupping treatment*) OR abstract:(cupping treatment*)) OR (title:(therapy, cupping) OR abstract:(therapy, cupping)) OR (title:(treatment, cupping) OR abstract:(treatment, cupping)) OR (title:(scrapping) OR abstract:(scrapping)) OR (title:(scraping) OR abstract:(scraping)) OR (title:(Tai Ji) OR abstract:(Tai Ji)) OR (title:(Taiji*) OR abstract:(Taiji*)) OR (title:(T?ai Chi) OR abstract:(T?ai Chi)) OR (title:(Ji Quan, Tai) OR abstract:(Ji Quan, Tai)) OR (title:(Tai Ji Quan) OR abstract:(Tai Ji Quan)) OR (title:(Quan, Tai Ji) OR abstract:(Quan, Tai Ji)) OR (title:(Qigong) OR abstract:(Qigong)) OR (title:(Qi Gong) OR abstract:(Qi Gong)) OR (title:(Ch'i Kung) OR abstract:(Ch'i Kung)) OR (title:(Baduanjin) OR abstract:(Baduanjin)) OR (title:(Baduanjin exercise) OR abstract:(Baduanjin exercise)) OR (title:(eight-section* exercise) OR abstract:(eight-section* exercise)) OR (title:(Diet Therapy) OR abstract:(Diet Therapy)) OR (title:(diet* modification*) OR abstract:(diet* modification*)) OR (title:(modification, diet*) OR abstract:(modification, diet*)) OR (title:(diet therap*) OR abstract:(diet therap*)) OR (title:(therapy, diet) OR abstract:(therapy, diet)) OR (title:(Psychotherap*) OR abstract:(Psychotherap*)) OR (title:(Music Therapy) OR abstract:(Music Therapy)) OR (title:(Therapy, Music) OR abstract:(Therapy, Music))) AND ((title:(coronary disease*) OR abstract:(coronary disease*)) OR (title:(coronary heart disease*) OR abstract:(coronary heart disease*)) OR (title:(disease*, coronary) OR abstract:(disease*, coronary)) OR (title:(disease*, coronary heart) OR abstract:(disease*, coronary heart)) OR (title:(heart disease*, coronary) OR abstract:(heart disease*, coronary)) OR (title:(angina pectoris) OR abstract:(angina pectoris)) OR (title:(angor pectoris) OR abstract:(angor pectoris)) OR (title:(stenocardia*) OR abstract:(stenocardia*)) OR (title:(myocardial infarct*) OR abstract:(myocardial infarct*)) OR (title:(infarct*, myocardial) OR abstract:(infarct*, myocardial)) OR (title:(stroke*, cardiovascular) OR abstract:(stroke*, cardiovascular)) OR (title:(cardiovascular stroke*) OR abstract:(cardiovascular stroke*)) OR (title:(heart attack*) OR abstract:(heart attack*)))

Appendix A5: Search Strategy for EBSCO (AMED, CINAHL, PsycINFO)

#	Query	Limiters/Expanders
S1	SU coronary disease	Expanders - Apply equivalent subjects Search modes - Boolean/Phrase
S2	TI coronary diseases OR AB coronary diseases OR TI coronary heart disease* OR AB coronary heart disease* OR TI disease*, coronary OR AB disease*, coronary OR TI disease*, coronary heart OR AB disease*, coronary heart OR TI heart disease*, coronary OR AB heart disease*, coronary	Limiters - Linked Full Text Expanders - Apply equivalent subjects Search modes - Boolean/Phrase
S3	SU Angina Pectoris	Limiters - Linked Full Text Expanders - Apply equivalent subjects Search modes - Boolean/Phrase
S4	TI angor pectoris OR AB angor pectoris OR TI stenocardia* OR AB stenocardia*	Limiters - Linked Full Text Expanders - Apply equivalent subjects Search modes - Boolean/Phrase
S5	SU Myocardial Infarction	Limiters - Linked Full Text Expanders - Apply equivalent subjects Search modes - Boolean/Phrase
S6	TI infarct*, myocardial OR AB infarct*, myocardial OR TI stroke*, cardiovascular OR AB stroke*, cardiovascular OR TI cardiovascular stroke* OR AB cardiovascular stroke* OR TI myocardial infarct* OR AB myocardial infarct* OR TI heart attack* OR AB heart attack*	Limiters - Linked Full Text Expanders - Apply equivalent subjects Search modes - Boolean/Phrase
S7	S1 OR S2 OR S3 OR S4 OR S5 OR S6	Limiters - Linked Full Text Expanders - Apply equivalent subjects Search modes - Boolean/Phrase
S8	SU Acupuncture Therapy	Limiters - Linked Full Text Expanders - Apply equivalent subjects Search modes - Boolean/Phrase
S9	TI treatment*, acupuncture OR AB treatment*, acupuncture OR TI acupuncture treatment* OR AB acupuncture treatment* OR TI therap*, acupuncture OR AB therap*, acupuncture OR TI acupotom* OR AB acupotom*	Limiters - Linked Full Text Expanders - Apply equivalent subjects Search modes - Boolean/Phrase
S10	SU Acupuncture, Ear	Limiters - Linked Full Text Expanders - Apply equivalent subjects Search modes - Boolean/Phrase
S11	TI ear acupuncture* OR AB ear acupuncture* OR TI acupuncture*, auricular OR AB acupuncture*, auricular OR TI auricular acupuncture* OR AB auricular acupuncture* OR TI acupuncture*, ear OR AB acupuncture*, ear	Limiters - Linked Full Text Expanders - Apply equivalent subjects Search modes - Boolean/Phrase
S12	SU Electroacupuncture	Limiters - Linked Full Text Expanders - Apply equivalent subjects Search modes - Boolean/Phrase
S13	TI electroacupuncture OR AB electroacupuncture	Limiters - Linked Full Text Expanders - Apply equivalent subjects Search modes - Boolean/Phrase
S14	SU Moxibustion	Limiters - Linked Full Text Expanders - Apply equivalent subjects Search modes - Boolean/Phrase
S15	TI moxabustion OR AB moxabustion	Limiters - Linked Full Text Expanders - Apply equivalent subjects Search modes - Boolean/Phrase
S16	SU Massage	Limiters - Linked Full Text Expanders - Apply equivalent subjects Search modes - Boolean/Phrase
S17	TI therap*, zone OR AB therap*, zone OR TI zone therap* OR AB zone therap* OR TI therap*, massage OR AB therap*, massage OR TI massage therap* OR AB massage therap* OR TI tuina OR AB tuina	Limiters - Linked Full Text Expanders - Apply equivalent subjects Search modes - Boolean/Phrase
S18	SU Cupping Therapy	Limiters - Linked Full Text Expanders - Apply equivalent subjects Search modes - Boolean/Phrase
S19	TI cupping treatment* OR AB cupping treatment* OR TI therapy, cupping OR AB therapy, cupping OR TI cupping therap* OR AB cupping therap* OR TI treatment, cupping OR AB treatment, cupping OR TI scrapping OR AB scrapping OR TI scraping OR AB scraping	Limiters - Linked Full Text Expanders - Apply equivalent subjects Search modes - Boolean/Phrase

S20	SU Tai Ji	Limiters - Linked Full Text Expanders - Apply equivalent subjects Search modes - Boolean/Phrase
S21	TI Taiji* OR AB Taiji* OR TI T?ai Chi OR AB T?ai Chi OR TI Ji Quan, Tai OR AB Ji Quan, Tai OR TI Tai Ji Quan OR AB Tai Ji Quan OR TI Quan, Tai Ji OR AB Quan, Tai Ji	Limiters - Linked Full Text Expanders - Apply equivalent subjects Search modes - Boolean/Phrase
S22	SU Qigong	Limiters - Linked Full Text Expanders - Apply equivalent subjects Search modes - Boolean/Phrase
S23	TI Qi Gong OR AB Qi Gong OR TI Ch'i Kung OR AB Ch'i Kung	Limiters - Linked Full Text Expanders - Apply equivalent subjects Search modes - Boolean/Phrase
S24	TI Baduanjin OR AB Baduanjin OR TI Baduanjin exercise OR AB Baduanjin exercise OR TI eight-section* exercise OR AB eight- section* exercise	Limiters - Linked Full Text Expanders - Apply equivalent subjects Search modes - Boolean/Phrase
S25	SU Diet Therapy	Limiters - Linked Full Text Expanders - Apply equivalent subjects Search modes - Boolean/Phrase
S26	TI diet* modification* OR AB diet* modification* OR TI modification, diet* OR AB modification, diet* OR TI diet therap* OR AB diet therap* OR TI therapy, diet OR AB therapy, diet	Limiters - Linked Full Text Expanders - Apply equivalent subjects Search modes - Boolean/Phrase
S27	SU Psychotherapy	Limiters - Linked Full Text Expanders - Apply equivalent subjects Search modes - Boolean/Phrase
S28	TI Psychotherap* OR AB Psychotherap*	Limiters - Linked Full Text Expanders - Apply equivalent subjects Search modes - Boolean/Phrase
S29	SU Music Therapy	Limiters - Linked Full Text Expanders - Apply equivalent subjects Search modes - Boolean/Phrase
S30	TI Therapy, Music OR AB Therapy, Music	Limiters - Linked Full Text Expanders - Apply equivalent subjects Search modes - Boolean/Phrase
S31	S8 OR S9 OR S10 OR S11 OR S12 OR S13 OR S14 OR S15 OR S16 OR S17 OR S18 OR S19 OR S20 OR S21 OR S22 OR S23 OR S24 OR S25 OR S26 OR S27 OR S28 OR S29 OR S30	Limiters - Linked Full Text Expanders - Apply equivalent subjects Search modes - Boolean/Phrase
S32	S7 AND S31	Limiters - Linked Full Text Expanders - Apply equivalent subjects Search modes - Boolean/Phrase
S33	S7 AND S31	Limiters - Linked Full Text Expanders - Apply equivalent subjects Narrow by Methodology: - meta analysis; Narrow by Methodology: - systematic review; Narrow by Methodology: - literature review Search modes - Boolean/Phrase

Appendix A6: Search Strategy for PROSPERO

Line	Search for	Hits
#1	Acupuncture	3028
#2	coronary disease	145
#3	coronary disease	145
#4	#1 AND #2	3
#5	Tai Ji	89
#6	#5 AND #2	2

#7	qigong	293
#8	Qigong	293
#9	#8 AND #2	0
#10	baduanjin	125
#11	#2 AND #10	1
#12	moxibustion	1075
#13	#12 AND #2	1
#14	Cupping	262
#15	#14 AND #2	0
#16	Massage	1045
#17	#16 AND #2	0
#18	scraping	46
#19	#18 AND #2	0
#20	Myocardial Infarction	3166
#21	Angina Pectoris	316
#22	Psychotherapy	1767
#23	Diet Therapy	143
#24	#23 OR #22 OR #18 OR #16 OR #14 OR #12 OR #10 OR #8 OR #5 OR #1	5691
#25	#21 OR #20 OR #2	3313
#26	#24 AND #25	95
#27	Music Therapy	374
#28	#27 OR #24	5931
#29	#28 AND #25	95

Appendix A7: Search Strategy for CNKI, Wanfang Database and SinoMed

CNKI

SU = (冠心病 + 冠状动脉粥样硬化性心脏病 + 心绞痛 + 心肌梗塞) * (针刺 + 针灸 + 电针 + 耳针 + 刮痧 + 推拿 + 按摩 + 拔罐 + 太极 + 太极拳 + 气功 + 八段锦 + 食疗 + 心理干预 + 音乐疗法) * (系统评价 + 系统综述 + meta 分析 + 荟萃分析) OR TKA = (冠心病 + 冠状动脉粥样硬化性心脏病 + 心绞痛 + 心肌梗塞) * (针刺 + 针灸 + 电针 + 耳针 + 刮痧 + 推拿 + 按摩 + 拔罐 + 太极 + 太极拳 + 气功 + 八段锦 + 食疗 + 心理干预 + 音乐疗法) * (系统评价 + 系统综述 + meta 分析 + 荟萃分析)

Wanfang Database

主题:((冠心病 OR 冠状动脉粥样硬化性心脏病 OR 心绞痛 OR 心肌梗塞) AND (针刺 OR 针灸 OR 电针 OR 耳针 OR 刮痧 OR 推拿 OR 按摩 OR 拔罐 OR 太极 OR 太极拳 OR 气功 OR 八段锦 OR 食疗 OR 心理干预 OR 音乐疗法 OR 中医情志) AND (系统评价 OR 系统综述 OR meta 分析 OR 荟萃分析)) or 题名或关键词:((冠心病 OR 冠状动脉粥样硬化性心脏病 OR 心绞痛 OR 心肌梗塞) AND (针刺 OR 针灸 OR 电针 OR 耳针 OR 刮痧 OR 推拿 OR 按摩 OR 拔罐 OR 太极 OR 太极拳 OR 气功 OR 八段锦 OR 食疗 OR 心理干预 OR 音乐疗法 OR 中医情志) AND (系统评价 OR 系统综述 OR meta 分析 OR 荟萃分析))

SinoMed

(("冠心病"[常用字段] OR "冠状动脉心脏病"[常用字段] OR "冠心病"[主题词]) OR ("冠状动脉疾病"[常用字段] OR "冠状动脉硬化"[常用字段] OR "冠状动脉动脉粥样硬化"[常用字段] OR "冠状动脉粥样硬化"[常用字段] OR "冠状动脉疾病"[主题词]) OR ("心绞痛"[常用字段] OR "心绞疼"[常用字段] OR "狭心症"[常用字段] OR "心绞痛"[主题词]) OR ("心肌梗死"[全部字段] OR "心脏病发作"[全部字段] OR "心肌梗塞"[全部字段] OR "心肌梗死"[主题词])) AND (("针刺"[常用字段] OR "药物针刺"[常用字段] OR "针刺"[主题词]) OR "针灸"[常用字段] OR ("电针"[常用字段] OR "电针"[主题词]) OR ("耳针"[常用字段] OR "耳针刺"[常用字段] OR "耳廓针刺"[常用字段] OR "耳廓针刺术"[常用字段] OR "针刺，耳"[主题词]) OR "刮痧"[常用字段] OR ("推拿"[常用字段] OR "推拿"[主题词]) OR ("按摩"[常用字段] OR "反射学"[常用字段] OR "体区疗法"[常用字段] OR "按摩"[主题词]) OR ("拔罐"[常用字段] OR "拔罐"[主题词]) OR ("太极"[常用字段] OR "太极(Tai Chi)"[常用字段] OR "太极(T'ai Chi)"[常用字段] OR "太极拳(Tai Ji Quan)"[常用字段] OR "太极(Taiji)"[常用字段] OR "太极(Tai-ji)"[常用字段] OR "太极拳(Taijiquan)"[常用字段] OR "太极"[主题词]) OR ("太极拳"[常用字段] OR "太极拳"[主题词]) OR ("气功"[常用字段] OR "气功"[主题词]) OR "八段锦"[常用字段] OR ("食疗"[常用字段] OR "食疗"[主题词]) OR "心理干预"[常用字段] OR ("音乐疗法"[全部字段] OR "音乐疗法"[主题词]) OR ("中医情志"[全部字段])) AND ("系统评价"[常用字段] OR "系统综述"[常用字段] OR "meta 分析"[常用字段] OR "荟萃分析"[常用字段])

Appendix A8: Search Strategy for Grey Literature sites

Google Scholar (http://scholar.google.com)

filetype:pdf intitle:("coronary disease" OR "coronary diseases" OR "coronary heart disease" OR "angina pectoris" OR "angor pectoris" OR "stenocardia" OR "myocardial infarction") AND intitle: ("systematic review" OR "meta-analysis" OR "systematic reviews")

GreyNet International(https://www.greynet.org)

(coronary disease* OR coronary heart disease* OR angina pectoris OR angor pectoris OR stenocardia OR myocardial infarction) AND (systematic review* OR meta-analysis)

Grey Literature Report (https://www.greylit.org)

coronary disease OR coronary heart disease OR angina pectoris OR angor pectoris OR stenocardia OR myocardial infarction

Appendix B: PRISMA results

Appendix B1: PRISMA 2020 for Abstracts results [1]

PRISMA (2020 Abstract) items			Xu W, 2021	Xu L, 2020	Zhang J, 2020	Luo N, 2020	Yang X, 2019	Zhou J, 2018	Chen Z, 2018	Li H, 2017	Li T, 2016	Zhang Z, 2015	Chen J, 2012	Nery RM, 2014	Yes		Partial Yes		No	
															n	%	n	%	n	%
Title	Title	1	PY	PY	PY	PY	PY	PY	Y	Y	Y	Y	Y	N	5	42	6	50	1	8
Background	Objectives	2	Y	Y	Y	Y	Y	Y	Y	Y	Y	Y	Y	Y	12	100	0	0	0	0
Methods	Eligibility criteria	3	PY	PY	PY	PY	PY	N	PY	PY	PY	N	PY	N	0	0	9	75	3	25
	Information sources	4	PY	Y	Y	PY	Y	Y	Y	N	Y	Y	Y	Y	9	75	2	17	1	8
	Risk of bias	5	N	N	N	Y	N	N	N	N	N	N	N	N	1	8	0	0	11	92
	Synthesis of results	6	Y	Y	Y	Y	Y	N	Y	Y	Y	N	Y	N	9	75	0	0	3	25
Results	Included studies	7	PY	PY	PY	Y	PY	PY	PY	PY	PY	PY	PY	PY	1	8	11	92	0	0
	Synthesis of results	8	Y	PY	PY	PY	Y	N	Y	PY	Y	PY	Y	PY	5	42	6	50	1	8
Discussion	Limitations of evidence	9	N	Y	Y	N	N	PY	Y	N	Y	N	Y	Y	6	50	1	8	5	42
	Interpretation	10	Y	Y	Y	Y	Y	Y	Y	Y	Y	Y	Y	Y	12	100	0	0	0	0
Other	Funding	11	N	N	N	N	N	N	N	N	N	N	N	N	0	0	0	0	12	100
	Registration	12	N	N	N	N	N	N	N	N	N	N	N	N	0	0	0	0	12	100

Appendix B2: PRISMA 2020 results [1]

PRISMA Items			Xu W, 2021	Xu L, 2020	Zhang J, 2020	Luo N, 2020	Yang X, 2019	Zhou J, 2018	Chen Z, 2018	Li H, 2017	Li T, 2016	Zhang Z, 2015	Chen J, 2012	Nery RM, 2014	Yes		Partial Yes		No		Not applicable	
															n	%	n	%	n	%	n	%
Title	Title	1	PY	PY	PY	PY	PY	PY	Y	Y	Y	Y	Y	N	5	42	6	50	1	8	0	0
Abstract	Abstract	2	PY	PY	PY	PY	PY	PY	PY	PY	PY	PY	PY	PY	0	0	12	100	0	0	0	0
Introduction	Rationale	3	Y	Y	Y	Y	Y	Y	Y	PY	Y	Y	Y	Y	11	92	1	8	0	0	0	0
	Objectives	4	Y	Y	Y	Y	Y	PY	Y	Y	Y	Y	Y	Y	11	92	1	8	0	0	0	0
Methods	Eligibility criteria	5	PY	PY	PY	PY	PY	PY	PY	PY	PY	PY	PY	PY	0	0	12	100	0	0	0	0
	Information sources	6	Y	Y	Y	Y	Y	Y	Y	Y	Y	Y	Y	Y	12	100	0	0	0	0	0	0
	Search strategy	7	PY	PY	PY	PY	PY	PY	PY	PY	PY	PY	PY	PY	0	0	12	100	0	0	0	0
	Selection process	8	Y	PY	Y	Y	Y	Y	Y	Y	Y	Y	Y	Y	11	92	1	8	0	0	0	0
	Data collection process	9	Y	PY	Y	Y	Y	Y	Y	Y	Y	Y	Y	Y	11	92	1	8	0	0	0	0
	Data items	10a	PY	PY	PY	N	N	PY	PY	N	PY	PY	PY	PY	0	0	9	75	3	25	0	0
		10b	PY	PY	PY	N	N	PY	PY	N	PY	PY	PY	PY	0	0	9	75	3	25	0	0
	Study risk of bias assessment	11	PY	Y	Y	Y	Y	Y	Y	Y	Y	Y	Y	Y	11	92	1	8	0	0	0	0
	Effect measures	12	Y	Y	Y	Y	Y	Y	Y	Y	Y	Y	Y	N	11	92	0	0	1	8	0	0
	Synthesis methods	13a	Y	Y	Y	Y	Y	Y	Y	Y	Y	Y	Y	N	11	92	0	0	1	8	0	0
		13b	Y	Y	Y	Y	Y	Y	Y	Y	Y	Y	Y	N	11	92	0	0	1	8	0	0
		13c	Y	Y	Y	Y	Y	Y	Y	Y	Y	Y	Y	N	11	92	0	0	1	8	0	0
		13d	Y	Y	Y	Y	Y	Y	Y	Y	Y	Y	Y	N	11	92	0	0	1	8	0	0
		13e	Y	Y	Y	Y	Y	Y	Y	Y	Y	Y	Y	N	11	92	0	0	1	8	0	0
		13f	Y	Y	Y	Y	Y	Y	Y	Y	Y	Y	Y	N	11	92	0	0	1	8	0	0
	Reporting bias assessment	14	N	N	N	Y	N	N	Y	Y	Y	N	N	N	4	33	0	0	8	67	0	0
	Certainty assessment	15	N	N	N	N	N	N	Y	N	N	N	N	N	1	8	0	0	11	92	0	0
Results	Study selection	16a	PY	Y	Y	N	Y	PY	PY	PY	PY	PY	Y	Y	5	42	6	50	1	8	0	0
		16b	N	N	N	N	N	N	N	N	N	N	N	N	0	0	0	0	12	100	0	0
	Study characteristics	17	Y	Y	Y	Y	Y	Y	Y	Y	Y	PY	Y	Y	11	92	1	8	0	0	0	0
	Risk of bias in studies	18	Y	Y	Y	Y	Y	PY	Y	Y	Y	PY	Y	Y	10	83	2	17	0	0	0	0

Results of individual studies	19	Y	Y	Y	Y	Y	Y	Y	Y	Y	Y	Y	PY	11	92	1	8	0	0	0	0

Appendix B2: PRISMA 2020 results (continued)

PRISMA Items			Xu W, 2021	Xu L, 2020	Zhang J, 2020	Luo N, 2020	Yang X, 2019	Zhou J, 2018	Chen Z, 2018	Li H, 2017	Li T, 2016	Zhang Z, 2015	Chen J, 2012	Nery RM, 2014	Yes n	Yes %	Partial Yes n	Partial Yes %	No n	No %	Not applicable n	Not applicable %
	Results of syntheses	20a	Y	Y	Y	Y	Y	Y	Y	Y	Y	Y	Y	Y	12	100	0	0	0	0	0	0
		20b	Y	Y	Y	Y	Y	Y	Y	Y	Y	Y	Y	NA	11	92	0	0	0	0	1	8
		20c	Y	Y	Y	Y	Y	Y	Y	Y	Y	Y	Y	NA	11	92	0	0	0	0	1	8
		20d	Y	Y	Y	Y	Y	Y	Y	Y	Y	Y	Y	NA	11	92	0	0	0	0	1	8
	Reporting biases	21	NA	N	NA	Y	NA	Y	Y	NA	Y	Y	Y	NA	6	50	0	0	1	8	5	42
	Certainty of evidence	22	N	N	N	N	N	N	Y	N	N	N	N	N	1	8	0	0	11	92	0	0
Discussion	Discussion	23a	Y	Y	Y	Y	Y	Y	Y	Y	Y	Y	Y	Y	12	100	0	0	0	0	0	0
		23b	Y	Y	Y	Y	Y	Y	Y	Y	Y	Y	Y	Y	12	100	0	0	0	0	0	0
		23c	Y	Y	Y	Y	Y	Y	Y	Y	Y	Y	Y	Y	12	100	0	0	0	0	0	0
		23d	Y	Y	Y	Y	Y	Y	Y	Y	Y	Y	Y	Y	12	100	0	0	0	0	0	0
Other information	Registration and protocol	24a	N	N	N	N	N	N	N	N	N	N	N	N	0	0	0	0	12	100	0	0
		24b	N	N	N	N	N	N	N	N	N	N	N	N	0	0	0	0	12	100	0	0
		24c	N	N	N	N	N	N	N	N	N	N	N	N	0	0	0	0	12	100	0	0
	Support	25	PY	PY	PY	N	PY	PY	N	PY	PY	PY	PY	Y	1	8	9	75	2	17	0	0
	Competing interests	26	N	N	N	N	N	N	N	N	N	N	N	Y	1	8	0	0	11	92	0	0
	Availability of data, code and other materials	27	N	N	N	N	N	N	N	N	N	N	N	N	0	0	0	0	12	100	0	0

Appendix C: AMSTAR2 results [2]

**Author, Year**	1. PICO components	2. A priori design	3. Rationale for study slection	4. Literature search	5. Duplicate Slection	6. Duplicate Extraction	7. List of excluded studies	8. Description of included studies	9. Risk of bias assessment in RCTs	10. Funding sources	11. Appropriate MA methods	12. Used RoB in MA	13. Used RoB in interpreting results	14. Discussion of heterogeneity	15. Pubication bias	16. Conflict of interest	**Overall Rating**
Xu W, 2021	Y	N	Y	N	Y	Y	N	Partial Y	Y	N	Y	Y	Y	Y	Y	N	Critically Low
Xu L, 2020	Y	N	Y	Partial Y	Y	Y	Partial Y	Partial Y	Y	N	Y	Y	Y	Y	Y	N	Low
Zhang J, 2020	Y	N	Y	Partial Y	Y	Y	N	Partial Y	Y	N	Y	Y	Y	Y	Y	N	Critically Low
Luo N, 2020	Y	N	Y	N	Y	N	N	Partial Y	Partial Y	N	Y	Y	Y	Y	Y	N	Critically Low
Yang X, 2019	Y	N	Y	Y	Y	N	N	Partial Y	Y	N	Y	Y	Y	Y	Y	N	Critically Low
Zhou J, 2018	Y	N	Y	N	Y	Y	N	Partial Y	Y	N	Y	Y	Y	Y	N	N	Critically Low
Chen Z, 2018	Y	N	Y	Partial Y	Y	Y	N	Partial Y	Y	N	Y	Y	Y	Y	Y	N	Critically Low
Li H, 2017	Y	N	Y	N	Y	Y	Partial Y	Partial Y	Partial Y	N	Y	Y	Y	Y	N	N	Critically Low
Li T, 2016	Y	N	Y	Partial Y	Y	Y	N	Partial Y	Partial Y	N	Y	Y	Y	Y	Y	N	Critically Low
Zhang Z, 2015	Y	N	Y	Partial Y	Y	Y	Partial Y	N	Y	N	Y	Y	Y	Y	Y	N	Low
Chen J, 2012	Y	N	Y	Partial Y	Y	Y	N	Partial Y	Y	N	Y	Y	Y	Y	Y	N	Critically Low
Nery RM, 2014	Y	N	Y	Partial Y	Y	Y	Partial Y	Partial Y	Y	N	Not applicable	Not applicable	Y	Y	Not applicable	Y	Low

Appendix D: Detailed Tables of Outcome Results [3]

Table D1. Acupuncture

Author, Year	Synthesis type	AMSTAR2 rating	Intervention and Sample size	# of studies	Pooled Estimates (MA) or Narrative Results (from SR with no MA)
CCEs					
Zhang Z, 2015	Systematic review	Low	Acupuncture plus usual care: 31<br>Usual care: 36	1	Acupuncture plus usual care resulted in more significant reduction in incidence of CCEs compared to usual care
Chen J, 2012	Meta-analysis	Critically Low	Acupuncture plus usual care: 70<br>Usual care: 70	2	Mean difference [95% CI]:<br>0.18 [0.04, 0.84] I-squared: 0%
Clinical effects (Total clinical efficiency: improvement rate of angina and ECG)					
Zhou J, 2018	Meta-analysis	Critically Low	Acupuncture plus usual care: 76<br>Usual care: 76	2	Mean difference [95% CI]:<br>6.01 [1.94, 18.66] I-squared: 0%
Clinical effects (improvement of angina symptoms)					
Zhou J, 2018	Systematic review	Critically Low	Acupuncture plus usual care: 15<br>Usual care: 15	1	Acupuncture plus usual care resulted in greater improvement of angina symptoms compared to usual care
Li H, 2017	Meta-analysis	Critically Low	Acupuncture plus usual care: 247<br>Usual care: 218	7	Mean difference [95% CI]:<br>6.31 [3.66, 10.87] I-squared: 0%
Zhang Z, 2015	Meta-analysis	Low	Acupuncture plus usual care: 490<br>Usual care: 426	9	Mean difference [95% CI]:<br>1.25 [1.17, 1.33] I-squared: 29%
Chen J, 2012	Meta-analysis	Critically Low	Acupuncture: 112<br>Usual care: 94	2	Mean difference [95% CI]:<br>3.59 [1.76, 7.29] I-squared: 0%
			Acupuncture plus usual care: 414<br>Usual care: 338	10	Mean difference [95% CI]:<br>4.23 [2.73, 6.56] I-squared: 0%
Clinical effects (improvement of ECG)					
Zhou J, 2018	Systematic review	Critically Low	Acupuncture plus usual care: 32<br>Usual care: 31	1	Acupuncture plus usual care resulted in no significant improvement of ECG compared to usual care
Li H, 2017	Meta-analysis	Critically Low	Acupuncture plus usual care: 163<br>Usual care: 130	5	Mean difference [95% CI]:<br>1.98 [1.18, 3.33] I-squared: 0%
Zhang Z, 2015	Meta-analysis	Low	Acupuncture plus usual care: 417<br>Usual care: 366	7	Mean difference [95% CI]:<br>1.28 [1.17, 1.39] I-squared: 0%
Chen J, 2012	Meta-analysis	Critically Low	Acupuncture: 112<br>Usual care: 94	2	Mean difference [95% CI]:<br>3.07 [1.54, 6.10] I-squared: 0%
			Acupuncture plus usual care: 411<br>Usual care: 336	10	Mean difference [95% CI]:<br>2.61 [1.83, 3.73] I-squared: 8%
Clinical effects (time to onset of angina relief in response to treatment)					
Chen J, 2012	Meta-analysis	Critically Low	Acupuncture: 72<br>Usual care: 56	1	Mean difference [95% CI]:<br>2.43 [1.63, 3.23]
			Acupuncture plus usual care: 61<br>Usual care: 61	2	Mean difference [95% CI]:<br>-1.40 [-1.10, -1.65] I-squared: 0%
SAS					
Zhou J, 2018	Systematic review	Critically Low	Acupuncture plus usual care: 15<br>Usual care: 15	1	Acupuncture plus usual care resulted in no significant difference in SAS compared to usual care

SDS					
**Author, Year**	**Synthesis type**	**AMSTAR2 rating**	**Intervention and Sample size**	**# of studies**	**Pooled Estimates (MA) or Narrative Results (from SR with no MA)**
Zhou J, 2018	Systematic review	Critically Low	Acupuncture plus usual care: 15<br>Usual care: 15	1	Acupuncture plus usual care resulted in no significant improvement of SDS compared to usual care
SAQ (total score)					
Zhou J, 2018	Systematic review	Critically Low	Acupuncture plus usual care: 15<br>Usual care: 15	1	Acupuncture plus usual care resulted in greater improvement of SAQ score compared to usual care
Adverse events related to the intervention					
Zhang Z, 2015	Meta-analysis	Low	Acupuncture plus usual care: 521<br>Usual care: 462	10	None of the studies reported any adverse effects associated with acupuncture therapy
Chen J, 2012	Meta-analysis	Critically Low	Acupuncture: 112<br>Usual care: 94	2	None of the studies reported any adverse effects associated with acupuncture therapy
			Acupuncture plus usual care: 476<br>Usual care: 397	12	

Table D2. Baduanjin

6MWT					
**Author, Year**	**Synthesis type**	**AMSTAR2 rating**	**Intervention and Sample size**	**# of studies**	**Pooled Estimates (MA) or Narrative Results (from SR with no MA)**
Xu W, 2021	Meta-analysis	Critically Low	Baduanjin plus usual care: 238<br>Usual care: 236	5	Mean difference [95% CI]:<br>68.99 [24.72, 113.27] I-squared: 99%
Yang X, 2019	Meta-analysis	Critically Low	Baduanjin plus usual care: 162<br>Usual care: 159	3	Mean difference [95% CI]:<br>51.38 [22.79, 79.98] I-squared: 93%
METs					
Yang X, 2019	Meta-analysis	Critically Low	Baduanjin plus usual care: 116<br>Usual care: 116	3	Mean difference [95% CI]:<br>0.54 [-0.07, 1.14] I-squared: 91%
SAS					
Xu W, 2021	Meta-analysis	Critically Low	Baduanjin plus usual care: 255<br>Usual care: 255	4	Mean difference [95% CI]:<br>-4.42 [-5.38, -3.46] I-squared: 5%
Xu L, 2020	Meta-analysis	Low	Baduanjin plus usual care: 155<br>Usual care: 155	3	Mean difference [95% CI]:<br>-6.10 [-7.24, -4.96] I-squared: 0%
Luo N, 2020	Meta-analysis	Critically Low	Baduanjin plus usual care: 435<br>Usual care: 435	8	Mean difference [95% CI]:<br>-6.44 [-8.75, -4.13] I-squared: 90%
SDS					
Xu W, 2021	Meta-analysis	Critically Low	Baduanjin plus usual care: 255<br>Usual care: 255	4	Mean difference [95% CI]:<br>-3.95 [-4.81, -3.09] I-squared: 28%
Xu L, 2020	Meta-analysis	Low	Baduanjin plus usual care: 95<br>Usual care: 95	2	Mean difference [95% CI]:<br>-6.25 [-8.52, -3.97] I-squared: 82%
Luo N, 2020	Meta-analysis	Critically Low	Baduanjin plus usual care: 325<br>Usual care: 325	6	Mean difference [95% CI]:<br>-5.25 [-7.10, -3.40] I-squared: 86%

SAQ (physical limitation)					
Xu L, 2020	Meta-analysis	Low	Baduanjin plus usual care: 125<br>Usual care: 125	3	Mean difference [95% CI]: 3.39 [0.39, 6.40] I-squared: 82%
			Baduanjin: 30<br>Usual medical exercise: 30	1	
Luo N, 2020	Meta-analysis	Critically Low	Baduanjin plus usual care: 258<br>Usual care: 259	8	Mean difference [95% CI]: 3.06 [1.27, 4.84] I-squared: 64%
			Baduanjin: 30<br>Usual medical exercise: 30	1	
SAQ (angina frequency)					
Xu L, 2020	Meta-analysis	Low	Baduanjin plus usual care: 125<br>Usual care: 125	3	Mean difference [95% CI]: 6.23 [1.31, 11.5] I-squared: 90%
			Baduanjin: 30<br>Usual medical exercise: 30	1	
Luo N, 2020	Meta-analysis	Critically Low	Baduanjin plus usual care: 408<br>Usual care: 409	9	Mean difference [95% CI]: 8.03 [6.85, 9.21] I-squared: 37%
			Baduanjin: 30<br>Usual medical exercise: 30	1	
SAQ (angina stability)					
Xu L, 2020	Meta-analysis	Low	Baduanjin plus usual care: 155<br>Usual care: 155	4	Mean difference [95% CI]: 12.22 [10.98，13.46] I-squared: 29%
Luo N, 2020	Meta-analysis	Critically Low	Baduanjin plus usual care: 408<br>Usual care: 409	9	Mean difference [95% CI]: 10.21 [7.44, 12.98] I-squared: 63%
			Baduanjin: 30<br>Usual medical exercise: 30	1	
SAQ (treatment satisfaction)					
Xu L, 2020	Meta-analysis	Low	Baduanjin plus usual care: 155<br>Usual care: 155	4	Mean difference [95% CI]: 1.43 [-0.74, 3.61] I-squared: 59%
			Baduanjin: 30<br>Usual medical exercise: 30	1	
Luo N, 2020	Meta-analysis	Critically Low	Baduanjin plus usual care: 408<br>Usual care: 409	9	Mean difference [95% CI]: 2.16 [-0.11, 4.43] I-squared: 69%
			Baduanjin: 30<br>Usual medical exercise: 30	1	
SAQ (disease perception)					
Xu L, 2020	Meta-analysis	Low	Baduanjin plus usual care: 155<br>Usual care: 155	4	Mean difference [95% CI]: 6.98 [3.61, 10.34] I-squared: 64%
			Baduanjin: 30<br>Usual medical exercise: 30	1	
Luo N, 2020	Meta-analysis	Critically Low	Baduanjin plus usual care: 408<br>Usual care: 409	9	Mean difference [95% CI]: 7.52 [5.03, 10.00] I-squared: 62%
			Baduanjin: 30<br>Usual medical exercise: 30	1	
Adverse events related to the intervention					

Luo N, 2020	Systematic review	Critically Low	Baduanjin plus usual care: 603<br>Usual care: 604	13	Adverse events related to Baduanjin were not found in the intervention group across included studies, which shows that Baduanjin is safe to be implemented in patients with CHD.
			Baduanjin: 30<br>Usual medical exercise: 30	1	

Table D3. Taiji

Health-related quality of life (SF-36 or MLHFQ)

Author, Year	Synthesis type	AMSTAR2 rating	Intervention and Sample size	# of studies	Pooled Estimates (MA) or Narrative Results (from SR with no MA)
Chen Z, 2018	Meta-analysis	Critically Low	Taiji plus jogging: 30<br>Jogging: 30	1	Mean difference [95% CI]:<br>0.79 [0.53, 1.05] I-squared: 0%
			Taiji plus usual care: 96<br>Usual care: 96	2	
Exercise tolerance assessment (VO2peak)					
Nery RM, 2014	Systematic review	Low	Taichi Chuan: 10<br>Physical activity counseling: 10	1	Taichi Chuan resulted in no significant difference in the improvement of VO2peak compared to physical activity counseling

Table D4. TCM exercise therapies

CCEs

Author, Year	Synthesis type	AMSTAR2 rating	Intervention and Sample size	# of studies	Pooled Estimates (MA) or Narrative Results (from SR with no MA)
Zhang J, 2020	Systematic review	Critically Low	TCM exercise therapies plus usual care: 50<br>Usual care: 50	1	TCM exercise therapies plus usual care resulted in more significant reduction in incidence of CCEs compared to usual care
Clinical effects (improvement of angina symptoms)					
Zhang J, 2020	Meta-analysis	Critically Low	TCM exercise therapies plus usual care: 142<br>Usual care: 140	2	Mean difference [95% CI]:<br>-1.13 [-0.56, 1.70] I-squared: 64%
Exercise tolerance assessment (VO2peak)					
Zhang J, 2020	Meta-analysis	Critically Low	TCM exercise therapies plus usual care: 91<br>Usual care: 95	2	Mean difference [95% CI]:<br>0.80 [-0.13, 1.72] I-squared: 87%
Exercise tolerance assessment (VO2/HR)					
Zhang J, 2020	Meta-analysis	Critically Low	TCM exercise therapies plus usual care: 91<br>Usual care: 95	2	Mean difference [95% CI]:<br>0.59 [0.10, 1.09] I-squared: 59%
SAS					
Zhang J, 2020	Meta-analysis	Critically Low	TCM exercise therapies plus usual care: 75<br>Usual care: 75	3	Mean difference [95% CI]:<br>-1.44 [-2.79, -0.10] I-squared: 92%

SDS					
Zhang J, 2020	Meta-analysis	Critically Low	TCM exercise therapies plus usual care: 75<br>Usual care: 75	3	Mean difference [95% CI]: -1.22[-2.03, -0.42] I-squared: 80%
SAQ (physical limitation)					
Zhang J, 2020	Meta-analysis	Critically Low	TCM exercise therapies plus usual care: 172<br>Usual care: 176	4	Mean difference [95% CI]: 0.48[0.14, 0.82] I-squared: 58%
SAQ (angina frequency)					
Zhang J, 2020	Meta-analysis	Critically Low	TCM exercise therapies plus usual care: 172<br>Usual care: 176	4	Mean difference [95% CI]: 0.64[0.43, 0.86] I-squared: 0%
SAQ (angina stability)					
Zhang J, 2020	Meta-analysis	Critically Low	TCM exercise therapies plus usual care: 172<br>Usual care: 176	4	Mean difference [95% CI]: 0.55[0.20, 0.90] I-squared: 60%
SAQ (treatment satisfaction)					
Zhang J, 2020	Meta-analysis	Critically Low	TCM exercise therapies plus usual care: 172<br>Usual care: 176	4	Mean difference [95% CI]: 0.70[0.07, 1.33] I-squared: 87%
SAQ (disease perception)					
Zhang J, 2020	Meta-analysis	Critically Low	TCM exercise therapies plus usual care: 172<br>Usual care: 176	4	Mean difference [95% CI]: 0.60[-0.11, 1.30] I-squared: 90%

Table D5. TCM emotional therapy

Clinical effects (total clinical efficiency:improvements of angina and ECG)

**Author, Year**	**Synthesis type**	**AMSTAR2 rating**	**Intervention and Sample size**	**# of studies**	**Pooled Estimates (MA) or Narrative Results (from SR with no MA)**
Li T, 2016	Meta-analysis	Critically Low	TCM emotional therapy plus usual care: 322<br>Usual care: 307	7	Mean difference [95% CI]: 0.21 [0.15, 0.27] I-squared: 18%
HAMD					
Li T, 2016	Meta-analysis	Critically Low	TCM emotional therapy plus usual care: 135<br>Usual care: 135	4	Mean difference [95% CI]: 6.64 [5.11, 8.18] I-squared: 8%
SAS					
Li T, 2016	Meta-analysis	Critically Low	TCM emotional therapy plus usual care: 97<br>Usual care: 89	3	Mean difference [95% CI]: 8.23 [6.79, 9.68] I-squared: 0%
SDS					
Li T, 2016	Meta-analysis	Critically Low	TCM emotional therapy plus usual care: 97<br>Usual care: 89	3	Mean difference [95% CI]: 8.27 [4.89, 11.66] I-squared: 77%

Appendix E: Tables with the overlap in the primary studies included in relevant reviews [3]

Table E1. Primary studies across included systematic reviews that examined Acupuncture

	Zhou J, 2018	Li H, 2017	Zhang Z, 2015	Chen J, 2012	Times Cited	Outcome(s)
Liu W, 2003	√			√	2	Clinical effects (improvements of ECG)
Qiu Y, 2013	√				1	Clinical effects (total clinical efficiency)
Yang Y, 2015	√				1	Clinical effects (total clinical efficiency)
Wang M, 2015	√				1	Clinical effects (improvements of angina symptoms), 6WMT, SAS, SDS, SAQ
Liu G, 2014		√			1	Clinical effects (improvements of angina symptoms and ECG)
Liu J, 2007		√	√		2	Clinical effects (improvements of angina symptoms and ECG)
Liu L, 2007		√			1	Clinical effects (improvements of angina symptoms)
Li X, 2015		√			1	Clinical effects (improvements of angina symptoms and ECG)
Wang X, 2000		√		√	2	Clinical effects (improvements of angina symptoms and ECG)
Xie Z, 2012		√	√		2	CCEs, Clinical effects (improvements of angina symptoms)
Guo X, 2014		√			1	Clinical effects (improvements of angina symptoms and ECG)
Yu W, 2006			√		1	Clinical effects (improvements of angina symptoms and ECG)
Yu S, 2005			√		1	Clinical effects (improvements of angina symptoms)
Cao J, 2002			√	√	2	Clinical effects (improvements of angina symptoms and ECG)
Luo L, 2006			√		1	Clinical effects (improvements of angina symptoms and ECG)
Liu WP,2006			√	√	2	Clinical effects (improvements of angina symptoms and ECG)
Li Y, 2012			√		1	Clinical effects (improvements of angina symptoms and ECG)
Tong Y, 2005			√	√	2	Clinical effects (improvements of angina symptoms and ECG)
Han A, 1999			√		1	Clinical effects (improvements of angina symptoms)
Yuan Z,1999				√	1	Clinical effects (improvements of ECG)
Chang P, 2005				√	1	Clinical effects (improvements of ECG)
Xu F, 2005				√	1	CCEs(non-fatal myocardial infarction), Clinical effects (improvements of angina symptoms and ECG)
Yu S, 2005				√	1	Clinical effects (time to onset of angina relief in response to treatment and improvements of angina symptoms)
Li C, 2005				√	1	Clinical effects (improvements of angina symptoms and ECG)
Zhai W, 2007				√	1	CCEs(non-fatal myocardial infarction), Clinical effects (improvements of angina symptoms and ECG)
Zhou W, 2007				√	1	Clinical effects (time to onset of angina relief in response to treatment, improvements of angina symptoms and ECG)
Yin L, 2009				√	1	Clinical effects (improvements of angina symptoms and ECG)
Zhang L, 2011				√	1	Clinical effects (improvements of angina symptoms and ECG)

Table E2. Primary studies across included systematic reviews that examined Baduanjin

	Xu W, 2021	Xu L, 2020	Luo N, 2020	Yang X, 2019	Times Cited	Outcome(s)
Wang J, 2018	√			√	2	6MWT
Tang T, 2019	√				1	6MWT
Lin J, 2012	√		√		2	SAS, SDS，SAQ，Clinical effect (improvement of angina symptoms)
Chen X, 2019	√		√		2	SAS, SDS，SAQ
Yu M, 2018	√			√	2	6MWT
Wang R, 2016	√		√		2	SAS, SDS, SAQ
Wang JJ, 2019	√	√	√		3	SAS, SDS
Li R, 2017	√				1	6MWT
Xiong X, 2016	√			√	2	6MWT
Hu L, 2018		√	√		2	SAS, SDS, SAQ
Zhang Z, 2019		√	√		2	SAQ
Hua L, 2018		√	√		2	SAS
Gu F,2018		√	√		2	SAQ
Lin X, 2012		√	√		2	SAQ
Wang X, 2019		√	√		2	SAQ
Zhao C, 2017			√	√	2	VO2peak, VO2/HR, METs, SAQ
Wu Y, 2016			√		1	SAS, SDS
Zhang X, 2017			√		1	SAQ
Wang J, 2019			√		1	SAS
Shi X, 2017				√	1	METs

Table E3. Primary studies across included systematic reviews that examined Taiji

	Chen Z, 2018	Nery 2014	Times Cited	Outcome(s)
Sato 2010		√	1	VO2peak
Zhang S, 2011	√		1	MLHFQ
Wang X, 2013	√		1	SF-36
Ding F, 2013	√		1	SF-36

Table E4. Primary studies across included systematic reviews that examined TCM exercise therapies

	Zhang J, 2020	Times Cited	Outcome(s)
Lin J, 2012	√	1	SAS, SDS，SAQ，Clinical effects (improvements of angina symptoms)
Jiang H, 2018	√	1	VO2peak, VO2 / HR, SAQ
Zhang Y, 2018	√	1	SAS, SDS
Wei X, 2018	√	1	SAQ
Li J, 2018	√	1	CCEs
Li S, 2017	√	1	Clinical effects (improvements of angina symptoms)
Zhao C, 2017	√	1	VO2peak, VO2/HR, METs, SAQ
Wu Y, 2016	√	1	SAS, SDS

Table E5. Primary studies across included systematic reviews that examined TCM emotional therapy

	Li T, 2016 n = 14	Times Cited	Outcome(s)
Huang J, 2012	√	1	HAMD
Sun Z, 2011	√	1	SDS
Wang L, 2014	√	1	Clinical effects (total clinical efficiency)
Yang L, 2013	√	1	SAS, SDS
Li X, 2012	√	1	SDS
Wang A, 2002	√	1	Clinical effects (total clinical efficiency and improvements of angina symptoms and ECG)
Huang JQ, 2012	√	1	HAMD
Liu X, 2012	√	1	Clinical effects (total clinical efficiency)
Liu K, 2012	√	1	SAS, SDS
Ren L, 2014	√	1	SAS, SDS
Gu B, 2012	√	1	HAMD, Clinical effects (total clinical efficiency)
Zhou H, 2015	√	1	Clinical effects (total clinical efficiency)
Wang J, 2014	√	1	HAMD, Clinical effects (total clinical efficiency)
Xu W, 2013	√	1	Clinical effects (total clinical efficiency)

References:

1 M. J. Page, J. E. McKenzie, P. M. Bossuyt et al., "The PRISMA 2020 statement: An updated guideline for reporting systematic reviews," JOURNAL OF CLINICAL EPIDEMIOLOGY, vol. 134, no.,pp.178-89,2021.

2 B. J. Shea, B. C. Reeves, G. Wells et al., "AMSTAR 2: a critical appraisal tool for systematic reviews that include randomised or non-randomised studies of healthcare interventions, or both," BMJ, vol. 358, no.,pp. j4008,2017.

3 https://systematicreviewsjournal.biomedcentral.com/articles/10.1186/s13643-019-1163-9
