# Supplementary material for: Effects and Safety of Non-Pharmacological Therapies of Traditional Chinese Medicine for Coronary Heart Disease: An Overview of Systematic Reviews
Source: Evid Based Complement Alternat Med. 2022 Mar 19;2022:8465269. doi: 10.1155/2022/8465269 (PMC8957469; doi:10.1155/2022/8465269)
Supplement: Supplementary Materials — Supplementary Material 1: PRIO-harms checklist for this overview. Supplementary Material 2: the appendices include all supplemental data and information. Appendix A: Search Strategy for Database and Gray Literature. Appendix B: PRISMA Results. Appendix C: AMSTAR Results. Appendix D: Detailed Tables of Outcome Results. Appendix E: Tables with the overlap in the primary studies included in relevant reviews. Supplementary Material 3: The matrix of evidence about primary studies across all included reviews. [file 8465269.f1.zip › 8465269.f1/Additional file 3 (1).pdf]

Additional File 3 Matrix of Evidence [1]

	Xu W, 2021	Xu L, 2020	Zhang J, 2020	Luo N, 2020	Yang X, 2019	Zhou J, 2018	Chen Z, 2018	Li H, 2017	Li T, 2016	Zhang Z, 2015	Chen J, 2012	Nery RM, 2014	Times Cited	Outcome(s)
Wang J, 2018	√				√								3	6MWT
Tang T, 2019	√												1	6MWT
Lin J, 2012	√		√	√									3	SAS, SDS，SAQ，Clinical effects (improvements of angina symptoms)
Chen X, 2019	√			√									2	SAS, SDS
Yu M, 2018	√				√								3	6MWT
Wang R, 2016	√			√									2	SAS, SDS
Wang J, 2019	√	√		√									3	SAS, SDS
Li R, 2017	√												1	6MWT
Xiong X, 2016	√				√								3	6MWT
Hu L, 2018		√		√									2	SAS, SDS, SAQ
Zhang Z, 2019		√		√									2	SAQ
Hua L, 2018		√		√									2	SAS
Gu F, 2018		√		√									2	SAQ
Lin X, 2012		√		√									2	SAQ
Wang X, 2019		√		√									2	SAQ
Jiang H, 2018			√										1	VO2peak, VO2 / HR, SAQ
Zhang Y, 2018			√										1	SAS, SDS
Wei X, 2018			√										1	SAQ
Li J, 2018			√										1	CCEs
Li S, 2017			√										1	Clinical effects (improvements of angina symptoms)
Zhang C, 2017			√	√	√								3	VO2peak, VO2 / HR, METs, SAQ
Wu Y, 2016			√	√									2	SAS, SDS
Zhang X, 2017				√									1	SAQ
Wang J, 2019				√									1	SAS
Shi X, 2017					√								1	METs
Liu W, 2003						√					√		2	Clinical effects (improvements of ECG)
Qiu Y, 2013						√							1	Clinical effects (total clinical efficiency
Yang Y, 2015						√							1	Clinical effects (total clinical efficiency
Wang M, 2015						√							1	Clinical effects (improvements of angina symptoms), 6WMT, SAS, SDS, SAQ
Sato, 2010												√	1	VO2peak
Zhang S, 2011							√						1	MLHFQ
Wang X, 2013							√						1	SF-36
Ding F, 2013							√						1	SF-36
Liu G, 2014								√					1	Clinical effects (improvements of angina symptoms and ECG)

Liu J, 2007								√		√			2	Clinical effects (improvements of angina symptoms and ECG)
Liu L, 2007								√					1	Clinical effects (improvements of angina symptoms)
Li X, 2015								√					1	Clinical effects (improvements of angina symptoms and ECG)
Wang X, 2000								√			√		2	Clinical effects (improvements of angina symptoms and ECG)
Xie Z, 2012								√		√			2	CCEs, Clinical effects (improvements of angina symptoms)
Guo X, 2014								√					1	Clinical effects (improvements of angina symptoms and ECG)
Huang J, 2012									√				1	HAMD
Sun Z, 2011									√				1	SDS
Wang L, 2014									√				1	Clinical effects (total clinical efficiency
Yang L, 2013									√				1	SAS, SDS
Li X, 2012									√				1	SDS
Wang A, 2002									√				1	Clinical effects (total clinical efficiency and improvements of angina symptoms and ECG)
Huang JQ, 2012									√				1	HAMD
Liu X, 2012									√				1	Clinical effects (total clinical efficiency
Liu K, 2012									√				1	SAS, SDS
Ren L, 2014									√				1	SAS, SDS
Gu B, 2012									√				1	HAMD, Clinical effects (total clinical efficiency)
Zhou H, 2015									√				1	Clinical effects (total clinical efficiency
Wang J, 2014									√				1	HAMD, Clinical effects (total clinical efficiency)
Xue W, 2013									√				1	Clinical effects (total clinical efficiency
Yu W, 2006										√			1	Clinical effects (improvements of angina symptoms and ECG)
Yu S, 2005										√			1	Clinical effects (improvements of angina symptoms)
Cao J, 2002										√	√		2	Clinical effects (improvements of angina symptoms and ECG)
Luo L, 2006										√			1	Clinical effects (improvements of angina symptoms and ECG)
Liu W, 2006										√	√		2	Clinical effects (improvements of angina symptoms and ECG)
Li Y, 2012										√			1	Clinical effects (improvements of angina symptoms and ECG)

Tong Y, 2005										√	√		2	Clinical effects (improvements of angina symptoms and ECG)
Han A, 1999										√			1	Clinical effects (improvements of angina symptoms)
Yuan Z, 1999											√		1	Clinical effects (improvements of ECG)
Chang P, 2005											√		1	Clinical effects (improvements of ECG)
Xu F, 2005											√		1	CCEs(non-fatal myocardial infarction), Clinical effects (improvements of angina symptoms and ECG)
Yu S, 2005											√		1	Clinical effects (time to onset of angina relief in response to treatment and improvements of angina symptoms)
Li C, 2005											√		1	Clinical effects (improvements of angina symptoms and ECG)
Zhai W, 2007											√		1	CCEs(non-fatal myocardial infarction), Clinical effects (improvements of angina symptoms and ECG)
Zhou W, 2007											√		1	Clinical effects (time to onset of angina relief in response to treatment, improvements of angina symptoms and ECG)
Yin L, 2009											√		1	Clinical effects (improvements of angina symptoms and ECG)
Zhang L, 2011											√		1	Clinical effects (improvements of angina symptoms and ECG)

CCA = (N−r)/((r*c)−r))

CCA = ((9+7+8+17+5+4+3+7+14+10+14+1) - 82)/ ((12*71) - 71)=28/ 781 = 0.0358

References

1 https://systematicreviewsjournal.biomedcentral.com/articles/10.1186/s13643-019-1163-9
